# Nab3’s localization to a nuclear granule in response to nutrient deprivation is determined by its essential prion-like domain

**DOI:** 10.1371/journal.pone.0209195

**Published:** 2018-12-17

**Authors:** Travis J. Loya, Thomas W. O’Rourke, William C. Simke, Joshua B. Kelley, Daniel Reines

**Affiliations:** 1 Department of Biochemistry, Emory University School of Medicine, Atlanta, Georgia, United States of America; 2 Department of Molecular and Biomedical Sciences, University of Maine, Orono, Maine, United States of America; University of Edinburgh, UNITED KINGDOM

## Abstract

Ribonucleoprotein (RNP) granules are higher order assemblies of RNA, RNA-binding proteins, and other proteins, that regulate the transcriptome and protect RNAs from environmental challenge. There is a diverse range of RNP granules, many cytoplasmic, which provide various levels of regulation of RNA metabolism. Here we present evidence that the yeast transcription termination factor, Nab3, is targeted to intranuclear granules in response to glucose starvation by Nab3’s proline/glutamine-rich, prion-like domain (PrLD) which can assemble into amyloid *in vitro*. Localization to the granule is reversible and sensitive to the chemical probe 1,6 hexanediol suggesting condensation is driven by phase separation. Nab3’s RNA recognition motif is also required for localization as seen for other PrLD-containing RNA-binding proteins that phase separate. Although the PrLD is necessary, it is not sufficient to localize to the granule. A heterologous PrLD that functionally replaces Nab3’s essential PrLD, directed localization to the nuclear granule, however a chimeric Nab3 molecule with a heterologous PrLD that cannot restore termination function or viability, does not form granules. The Nab3 nuclear granule shows properties similar to well characterized cytoplasmic compartments formed by phase separation, suggesting that, as seen for other elements of the transcription machinery, termination factor condensation is functionally important.

## Introduction

In yeast, transcription by RNA polymerase II can be terminated in two major ways: The Nrd1-Nab3-Sen1 (NNS) pathway which primarily generates short noncoding transcripts, or the polyadenylation-coupled termination pathway, where termination is associated with nascent transcript cleavage and polyadenylation of the RNA [1]. The NNS termination pathway contains Nrd1 and Nab3, essential RNA-binding proteins with canonical RNA recognition motifs (RRMs) [2–7]. Nab3 and Nrd1 heterodimerize and bind specific sequences in target RNAs, as well as interact with the C-terminal domain (CTD) of the large subunit of RNA polymerase II *via* Nrd1’s CTD-interacting domain [1]. Upon binding a target RNA, Nrd1-Nab3 recruits Sen1, a helicase responsible for termination of elongation [8–10]. The terminated transcript can be further processed by the TRAMP complex and the nuclear exosome.

**Fig 1:**
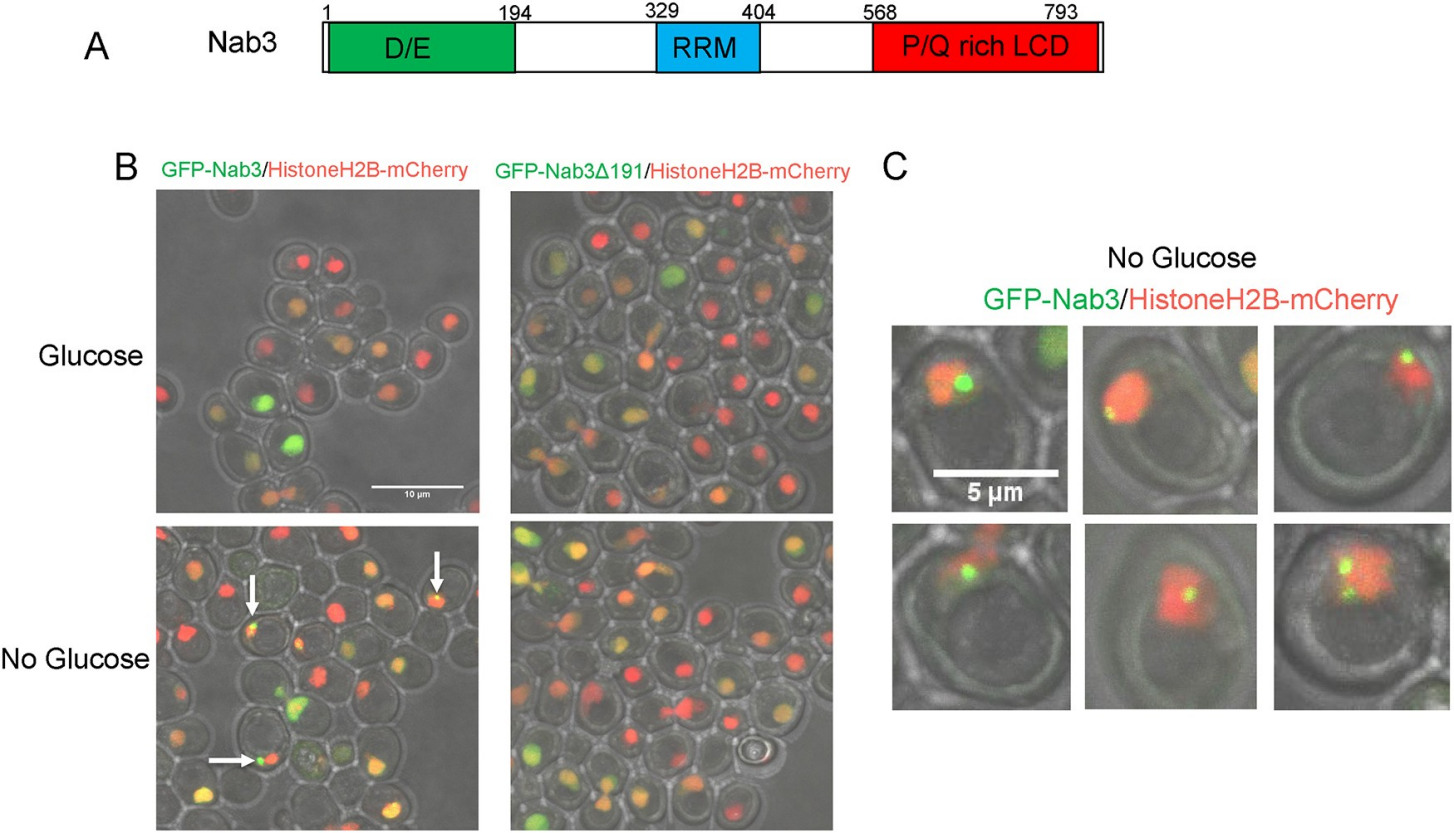
Re-localization of Nab3 to a granule during glucose starvation. A. Schematic of the domain organization of *S*. *cerevisiae* Nab3 showing the non-essential aspartate/glutamate rich domain (“D/E”), the RNA recognition motif (“RRM”), and the essential proline/glutamine rich low complexity domain defined as a PrLD by Alberti *et al*. [13] are shown. B. *S*. *cerevisiae* containing an integrated C-terminal mCherry-tagged histone H2B was transformed with an expression plasmid containing N-terminal GFP-tagged Nab3 (DY32309) or GFP-tagged Nab3Δ191 (DY3233). Cells were grown to log phase and washed into SC ura^-^ leu^-^ glucose-free or glucose-containing medium for two hours. Cells were placed on 1.5% agarose pads and Z-stacks were imaged using a confocal microscope. Maximum intensity projections of a field of cells show GFP-Nab3 forms granules only when glucose-starved (white arrows). C. Representative GFP-Nab3 granule-containing cells from a glucose-starved culture are shown.

Prior work in our lab has established that a C-terminal segment of Nab3 is essential for viability and plays a role in termination in both a reporter assay and at endogenous targets of NNS regulation [11, 12]. This Nab3 domain is scored as prion-like by a computer algorithm trained on prion sequences from yeast [13]. The prion-like domain (PrLD) of Nab3 is of low complexity with a skewed overrepresentation of glutamine (Q; 27%) and proline (P; 17%) (Fig 1A). It is relatively unstructured but can assemble into authentic amyloid polymers with a characteristic cross-beta architecture that further organizes into hydrogels. A purified portion of the PrLD, as well as full length Nab3, form amyloid filaments *in vitro* [12, 14]. Removal of even part of the PrLD from full-length Nab3 abrogates this property of the protein [14, 15]. A previously described derivative of the Nab3 PrLD in which glutamates are substituted for each of 24 glutamines, fails to polymerize *in vitro* and, when included in full-length Nab3, fails to support cell viability [15]. As well, exchanging Nab3’s PrLD for some, but not all, heterologous yeast PrLDs with amyloid polymerizing properties, can rescue cell viability and termination activity [16]. Thus, although a PrLD can polymerize in vitro, that property is not sufficient to rescue Nab3 function, leading to the idea that there are subcategories of PrLDs amongst RNA-binding proteins that govern protein activity. PrLDs are over-represented in RNA-binding proteins where they are important for supporting phase separation by their client protein as a means of assembling a subcellular compartment in which metabolic processes take place [17, 18]. Some of these subcellular compartments include the nucleolus and Cajal bodies in the nucleus, and P-bodies and stress granules in the cytoplasm, all of which are enriched in RNA-binding proteins and RNAs [19, 20]. Recently, RNA polymerase II and mediator have been shown to cluster in transcription-dependent phase-separated nuclear condensates in mammalian cells [21–25]. This suggests that many aspects of the biogenesis of mature RNAs from initiation to termination, may be governed by phase separation of the cognate machinery.

In addition to possessing a PrLD, other features of Nab3 are suggestive that it may operate *via* phase separation. Genetic and biochemical data support a model in which Nab3, and its dimerization partner Nrd1, function as multimers while binding with sequence-specificity to nascent RNA. The complex can bind RNA polymerase II through the CTD-interaction domain of Nrd1. These protein-protein and protein-nucleic acid interactions are important for the termination process [6, 12]. Thus, the function of the PrLD could serve as a device used to assemble a functional termination complex. The ability of some heterologous PrLDs to substitute for Nab3’s PrLD in supporting the protein’s termination function, as well as cell viability [16], suggests that the essential role played by Nab3’s PrLD is to facilitate the assembly of the termination factor into a specific polymeric complex. Indeed, Nab3 interacts with itself in living yeast when tested in a protein-protein interaction assay and in a yeast strain in which two different mutant *nab3* alleles can cross-complement [12, 14].

Changes in the transcriptome following glucose-deprivation are mediated in part by the NNS pathway. Here we exploit a recently discovered, glucose deprivation-induced, re-localization of Nab3 to a unique granule within the nucleus to learn if Nab3’s PrLD is involved in this process [26]. We show that truncation or a reduction in the glutamine-richness of the Nab3 PrLD prevented the recruitment of Nab3 to the granule when subjected to the glucose starvation paradigm. Though necessary, the PrLD was not sufficient to be recruited to the granule. Further characterization of granule formation revealed that it is dynamic; time lapse microscopy using microfluidics showed that minutes after the re-addition of glucose to starved cells, Nab3 rapidly delocalizes and becomes distributed throughout the nucleus. A functional Nab3 chimera employing a heterologous PrLD in place of its own, also reorganizes into the granule following a glucose-deprivation challenge while another heterologous PrLD that does not rescue Nab3 function, fails to localize to granules when substituted for Nab3’s own PrLD. These findings suggest that there is an important inducible rearrangement of Nab3 that resembles a phase separation event in which a new compartment can be reversibly assembled in yeast nuclei. This property relies upon on its essential PrLD.

## Materials and methods

### Plasmid construction

The pRS315GFPNab3 plasmid was generated (Genscript Inc.) by inserting eGFP after the start codon of the Nab3 open reading frame in pRS315Nab3. The pRS315GFPNab3Δ191 plasmid was generated from pRS315GFPNab3 by PCR deletion using oligonucleotides 5’–ACCATATGGCTGTTGCTG–3’ and 5’–TAGACTCCCTTTTTTCAATCTTTTCCATTTCTTG-3’. The *NdeI* site in GFP was deleted from plasmid pRS315GFPNab3 by PCR mutagenesis using the oligonucleotide 5’–CCCGGATCACATGAAACGGC–3’ and 5’-TATCTTGAAAAGCACTGAACACCATAA–3’ to generate pRS315GFPNab3NdeI. The plasmids pRS315GFPNab3Sup35 and pRS315GFPNab3Rat1 were constructed by releasing part of the PrLD coding sequence from pRS315GFPNab3NdeI with *Nde*I and *Xho*I followed by insertion of the heterologous PrLDs released from *Nde*I and *Xho*I cut pRS315Nab3Sup35 and PRS315Nab3Rat1, respectively. The plasmid pRS315-GFP-PrLD was created by PCR mutagenesis with the oligonucleotides 5’-AATTATGGGATGGGATGCCACCACC-3’ and 5’-GCAGCCGGATCCTTTGT-3’. pRS315yomKate2-Nab3 incorporated yeast-optimized mKate2 sequences [27] in frame at the N-terminus of Nab3 (Genscript, Inc.). The plasmid pRS315-GFPNab3ΔRRM was generated by PCR-based deletion using the oligonucleotides 5’-GACAACCCTCAAAGCGTTAG-3’ and 5’-ATTGTGCATCTCGGTGATTTT-3’ and the template pRS315-GFPNab3NdeI.

### Yeast strains

Yeast strains used in this study are presented in Table 1. Cells were grown in rich medium (YPD), synthetic medium (SC), or standard selective drop-out medium [SC ura^-^, SC leu^-^, or SC ura^-^ leu^-^; [28]] at 30°C unless otherwise indicated. Plasmids were transformed into yeast strains using the lithium acetate method [29]. DY3111 (Loya et al. 2017) was transformed with pRS315GFPNab3 and pRS315-Nab3Δ191 to yield yeast strains DY3205 and DY3183, respectively. DY3205 was cured of pRS316Nab3 by selection on 5-fluroorotic acid to create DY3206. HTB2 tagged with mCherry and marked with *HIS4* was amplified from genomic DNA of yeast strain YOL890 [gift from Dr. S. Wente] using 5’-GCAGCTGAACCAGCTTTACC-3’ and 5’-GCTTTCAGTCGAAAACAGC–3’. The PCR product was transformed into DY3206 using high efficiency lithium acetate transformation [30], transformants were isolated by growth on SC leu^-^ his^-^ media and verified by PCR using 5’-GGGAATGTTAAACCAGCTTTAGC-3’ and 5’-GCTTTCAGTCGAAAACAGC–3’ creating strain DY3228.

**Table 1:** 

Strain	Genotype	Reference
DY3111	*MATα ura3Δ0 his3Δ1 leu2Δ0 nab3Δ0*::*kanMX* [pRS316-*NAB3* (*URA3*)]	O’Rourke & Reines, 2016
DY3183	*MATα ura3Δ0 his3Δ1 leu2Δ0 nab3Δ0*::*kanMX* [pRS316-NAB3 (*URA3*)]	Loya & Reines, 2017
DY3205	*MATα ura3Δ0 his3Δ1 leu2Δ0 nab3Δ0*::*kanMX* [pRS316-NAB3 (URA3)] [pRS315GFPNab3 (*LEU2*)]	This Study
DY3206	*MATα ura3Δ0 his3Δ1 leu2Δ0 nab3Δ0*::*kanMX* [pRS315GFPNab3 (*LEU2*)]	This Study
DY3228	*MATα ura3Δ0 his3Δ1 leu2Δ0 nab3Δ0*::*kanMX htb2-mCherry*:*HIS3* [pRS315GFPNab3 (*LEU2*)]	This Study
DY3233	*MATα ura3Δ0 his3Δ1 leu2Δ0 nab3Δ0*::*kanMX htb2-mCherry*:*HIS3* [pRS316Nab3 (*URA3*) [pRS315GFPNab3*Δ*191 (*LEU2*)]	This Study
DY32309	*MATα ura3Δ0 his3Δ1 leu2Δ0 nab3Δ0*::*kanMX htb2-mCherry*:*HIS3* [pRS315GFPNab3 (*LEU2*)][pRS316Nab3 (*URA3*)]	This Study
DY32359	*MATα ura3Δ0 his3Δ1 leu2Δ0 nab3Δ0*::*kanMX htb2-mCherry*:*HIS3* [pRS316Nab3 (*URA3*)]	This Study
DY4525	*MATα ura3Δ0 his3Δ1 leu2Δ0 nab3Δ0*::*kanMX htb2-mCherry*:*HIS3* [pRS316Nab3 (*URA3*)] [pRS315GFPNab3Rat1 (*LEU2*)]	This Study
DY4530	*MATα his3Δ1 leu2Δ0 ura3Δ0 nab3Δ0*::*kanMX* [pRS315 yomkate2Nab3 (*LEU2*)]	This Study
DY4538	*MATa ura3Δ0 his3Δ1 leu2Δ0 btn2Δ0*::*kanMX* [pRS315 GFPNab (*LEU2*)]	This Study
DY4540	*MATa ura3Δ0 his3Δ1 leu2Δ0 nic96-GFP* [pRS315 yomkate2Nab3 (*LEU2*)]	This Study
DY4543	*MATa his3Δ1 leu2Δ0 met15Δ0 ura3Δ0 GFP-NAB3*::*HIS4*	This Study
DY4546	*MATα ura3Δ0 his3Δ1 leu2Δ0 nab3Δ0*::*kanMX htb2-mCherry*:*HIS3* [pRS316Nab3 (*URA3*)][pRS315GFPNab3Q—>E (*LEU2*)]	This Study
DY4549	*MATα ura3Δ0 his3Δ1 leu2Δ0 nab3Δ0*::*kanMX htb2-mCherry*:*HIS3* [pRS316Nab3 (*URA3*)] [pRS315-GFP-PrLD (*LEU2*)]	This Study
DY4551	*MATα ura3Δ0 his3Δ1 leu2Δ0 nab3Δ0*::*kanMX htb2-mCherry*:*HIS3* [pRS316Nab3 (*URA3*)][pRS315-GFP-Nab3ΔRRM (*LEU2*)]	This Study
DY4553	*MATα his3Δ1 leu2Δ0 ura3Δ0 nab3Δ0*::*kanMX* [pRS315 yomkate2Nab3 (*LEU2*)][pRS316GFPNab3 (*URA3*)]	This Study
YOL890	*ura3-1 his3-11*,*15 leu2-3*,*112 lys2 htb2-mCherry*:*HIS3*	Wente Lab

DY3228 was transformed with pRS316Nab3 to create DY3238 and grown in SC ura^-^ to encourage loss of pRS315GFPNab3. A derivative lacking this *LEU2*-marked plasmid was isolated yielding strain DY32359. DY32359 was transformed with pRS315GFPNab3, pRS315GFPNab3Δ191, pRS315GFPNab3Sup35, or pRS315Nab3Rat1 to create DY32309, DY3233, DY4524, and DY4525, respectively.

DY4540 was generated by transforming a strain containing chromosomal GFP-tagged *NIC96* (ThermoFisher) with pRS315-yomkate2-Nab3. DY4538 was generated by transforming a strain deleted for *BTN2* (courtesy of A. Corbett, Emory U.) with pRS315-GFP-Nab3. DY4546 was generated by transforming DY32359 with pRS315 Nab3Q—>E [15]. DY4549 was generated by transforming DY32359 with pRS315-GFP-PrLD. DY4551 was generated by transforming DY32359 with pRS315-GFPNab3ΔRRM.

For treatment with 1,6-hexanediol, glucose starved cells were adjusted to 5% 1,6-hexanediol and 10μg/ml digitonin as described [31]. Cells were incubated at 30°C for 30 min before confocal imaging.

Strain DY4543 was generated by allele replacement using pop-in/pop-out [32] to insert N-terminally GFP-tagged *NAB3* in its normal chromosomal location in yeast strain BY4741.

Strain DY4553 was generated from DY4530 by transforming it with pRS316GFPNab3Sup35. DY4530 was generated from DY3111 by transforming it with pRS315yomKate2Nab3 and chasing out the *URA3*-marked plasmid.

### Western blotting

Cells were grown in SC ura^-^ leu^-^ with or without glucose, as indicated. Cycloheximide (0.1%, Sigma-Aldrich) was added where indicated to a final concentration of 125μM for the indicated times. Cells were collected, washed with water, and boiled for 5 min in sample buffer [33], before being resolved on a 6% SDS-polyacrylamide gel. Proteins were electrophoretically transferred to nitrocellulose blocked with 5% nonfat dry milk [34], and probed with a mouse monoclonal antibody against Nab3 [2F12-2; Dr. M. Swanson [2]]. Signal was detected using horse radish peroxidase-conjugated anti-mouse IgG (Sigma Chemical Co.) and enhanced chemiluminescence was performed in 100mM Tris pH 8.5, 1.25 mM luminol, 200μM p-coumaric acid, and 0.01% H_2_O_2_.

### Confocal microscopy

Cells were grown overnight to mid-log phase in SC ura^-^ leu^-^ glucose media, washed three times in appropriate media for the experiment, and resuspended in the wash media. After 120 min of incubation, cells were deposited on 1.5% agarose pads. Confocal images were taken at room temperature using a confocal microscope (SP8; Leica) using a HC PL APO 63X 1.40 NA oil immersion lens, WD 0.14mm (Leica). Images were acquired using Leica Application Suite X 3.0.2.16120 (Leica). Representative cells are shown as maximum intensity projections or as single Z-planes as indicated from experiments repeated using three biological replicates. Images were processed using FIJI [35]. For counts of granules/cell, the three biological replicates were imaged for each condition and 100 to >1000 cells expressing GFP-Nab3 or its variants were counted by a blinded scorer. The exact number of GFP fusion-containing cells that were counted for each experimental condition is presented in Table 2. The number of cells with granules divided by the total number of cells with detectable pan-nuclear staining of that Nab3 derivative were multiplied by 100 to give the % with granules. Statistical significance was assessed using GraphPad Prism (v7) software and Fisher’s exact test.

**Table 2:** Number of cells counted for granule-content scoring.

Strain:	DY32309	DY3233	DY4538	DY4546	DY4551
relevant genotype:	*GFP-NAB3*	*GFP-nab3Δ191*	*Δbtn*	*GFP-nab3Q->E*	*GFP-nab3Δrrm*
glucose	1311	266	221	249	1145
glucose starvation	932	253	351	309	1725
sorbitol	849				
hexanediol	1473				
glucose 10 min	688				
sorbitol 10 min	509				
fructose10 min	100				
sucrose 10 min	100				
galactose 10 min	429				
galactose 12 hrs	1812				

### Microfluidics

Cultures were grown in SC ura^-^ leu^-^ to an OD_600_ between 0.2–0.6 at 30°C. Live-cell microfluidics experiments were performed using an IX83 (Olympus, Waltham MA) microscope with a Prime 95B CMOS Camera (Photometrics). Fluorescence and Differential Interference Contrast (DIC) images were acquired using an Olympus-APON-60X-TIRF objective. Five 290nm Z-stacks of GFP and RFP images were acquired using 3% intensity light from an Xcite 120 LEDBoost (Excelitas) with 35 ms and 50 ms exposure, respectively. Cells were imaged in a microfluidic device based on the Dial-a-wave design that allows for the rapid switching of media while holding the yeast in place [36, 37]. Glucose addition and removal was verified using AlexaFluor 647 dye (Life Technologies) present only in glucose-free media, imaged at 3% intensity for 100ms and 1 Z-slice. Cells were exposed to glucose for 1 hour, starved of glucose for 2 hours, and reintroduced to glucose in order to observe formation and dissolution of granules. Images were deconvolved using Huygens Software (Scientific Volume Imaging, Hilversum, Netherlands) Classic Maximum Likelihood Estimation (CMLE) Deconvolution Algorithm with a signal to noise ratio of 5. Masks of nuclei were made using ImageJ (National Institute of Health, Bethesda, MD) and data analysis was performed using MATLAB (MathWorks, Natick, MA).

### Quantification of granule formation

To quantify the fraction of granule formation, fluorescent intensities of the nucleus were determined for single cells through all time points. Fluorescent values were normalized to 8-bit scaling (0–255). The maximum and minimum fluorescent intensities for each cell were found through the time course for histogram normalization. Histograms were created from 0–255 with a bin width of five and normalized to sum to one. An average histogram was calculated for each cell during the first hour to serve as a baseline reading. Using MATLAB’s’ ‘fit’ function, a Gaussian curve was fit to each average histogram to acquire the mean of the histogram and the standard deviation. The MATLAB ‘fit’ function was used with ‘gauss1’ fit type and a ‘NonlinearLeastSquares’ method. Lower limits for the mean of the Gaussian were set to zero, as the fluorescence values cannot be negative. Granule formation involves an increase in fluorescence intensity from the baseline reading, as there is a higher concentration of GFP-Nab3 in each pixel. To measure the increased fluorescence derived from granule formation, we quantified the fraction of the fluorescence that was present in bins three standard deviations above the mean. We call this value the “spot fraction” and it represents the amount of fluorescence which registers as being in a granule, or other spot, by this method. We scored cells as positive for granule formation if more than two thirds of the total granule fraction measured occurred during glucose starvation.

## Results

### Nab3 localization to a granule is PrLD-dependent

**Fig 2:**
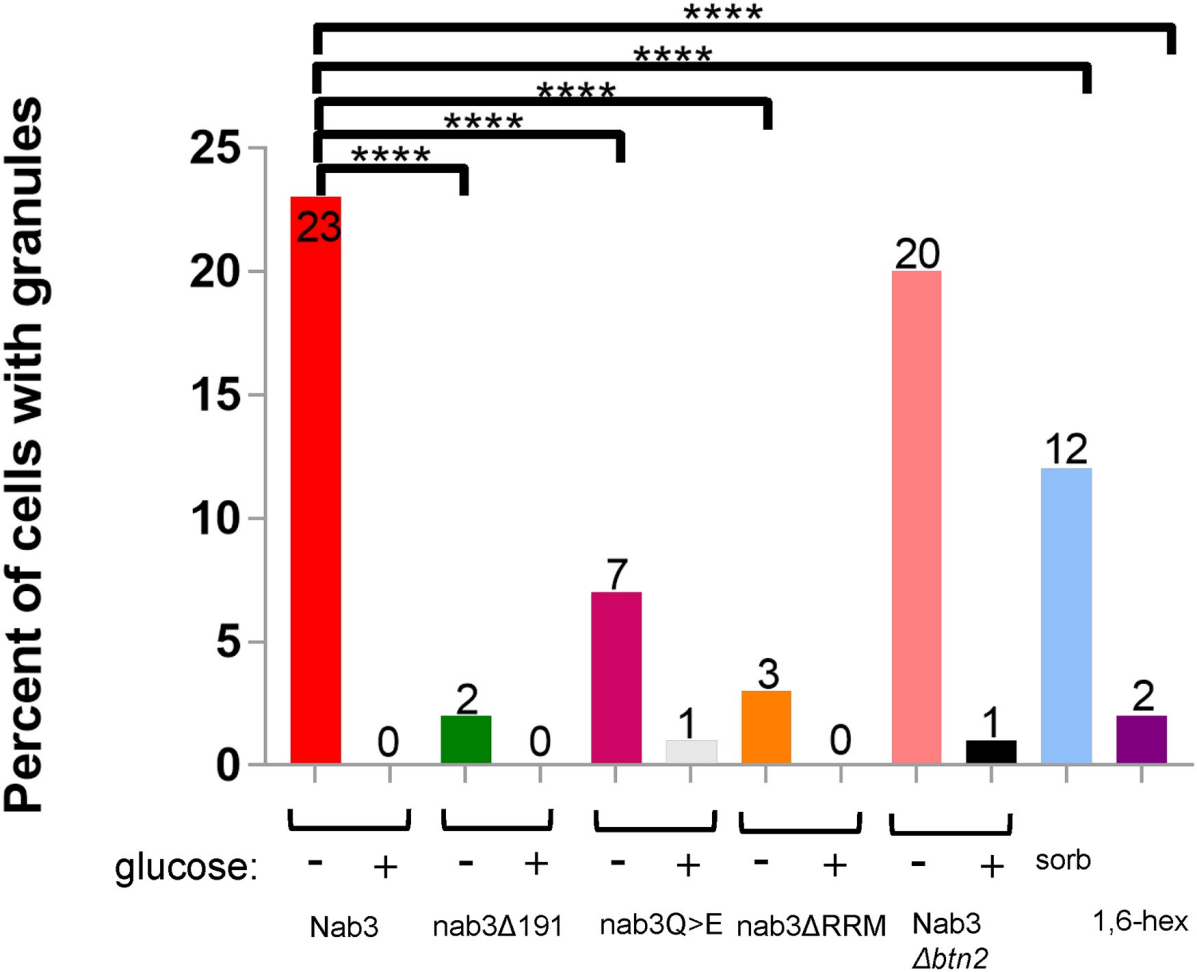
Quantification of granule-containing cells. *S*. *cerevisiae* strains were transformed with GFP tagged versions of Nab3 (DY32309), Nab3 lacking its C-terminal 191 residues, (Nab3Δ191; DY3233), Nab3 with a Q to E substituted PrLD (DY4546), or Nab3 deleted for its RRM motif (DY4551; ΔRRM). DY4538 had plasmid-borne GFP-Nab3 transformed into a *BTN2* deletion strain. Cells were washed into either glucose-free or glucose-containing media or sorbitol (“sorb”), as indicated, and incubated at 30°C for 2 hr, and imaged using confocal microscopy. For hexanediol treatment (“1,6 hex”, DY32309 was induced to form granules and incubated with the alcohol as described in Materials and Methods. Maximum intensity projections were created in FIJI and cells were binned into granule-containing or granule-free bins. Three biological replicates were used for imaging and counts are presented as the % of all cells expressing the respective GFP-fusion protein. The number of GFP-positive cells counted for each sample is presented in Table 2. The percent of cells containing granules were compared across groups under similar growth conditions using Fisher’s exact test. P-values of <0.0001 are indicated by ****.

Glucose restriction is commonly used to provoke the assembly of inducible compartments such as stress granules. In response to glucose starvation, Nrd1 and Nab3 shift from a diffuse pan-nuclear distribution to a concentrated perinuclear granule of unknown function [26]. Numerous studies have shown that low complexity PrLDs are often critical for proper localization of RNA-binding proteins to granules involved in the stress response, protein homeostasis, RNA metabolism, and development [38–40]. Thus, we asked: Is Nab3’s well-studied PrLD responsible for its ability to re-localize in response to carbon source restriction? To test this model, a series of *CEN* plasmid-borne, N-terminally GFP-tagged Nab3 variants were introduced into cells. Since many of the mutations are lethal, cells also expressed untagged Nab3 on a differently marked *CEN* plasmid. All Nab3 variants were expressed from the endogenous *NAB3* promoter in a strain deleted for chromosomal *NAB3*. Due to occasional plasmid loss [11, 41, 42], there was some heterogeneity from cell to cell in expression of the Nab3 proteins. To mark the nucleus, strains also included a chromosomal copy of mCherry-tagged histone H2B [43]. Cells were grown overnight to mid-log phase and washed free of glucose. As a control, an aliquot of cells was resuspended in fresh glucose-containing medium. After two hours, cells were imaged *via* confocal microscopy to assess the distribution of fluorescent protein. GFP-tagged wild type Nab3 exhibited a pan-nuclear distribution in glucose rich conditions. In glucose starved cells, Nab3 localized to a granule in roughly 20% of the population in which GFP-Nab3 was expressed, consistent with a previous report [26] [white arrows, Fig 1B; quantified in Fig 2]. This is comparable to the fraction of cells displaying granules when a *GFP-NAB3* allele is integrated into, and expressed from, its native chromosomal location (S1 Fig and [26]). This frequency is also similar to the fraction of cells that acquire cytoplasmic stress granules following glucose-restriction [44, 45]. If starvation-induced recruitment of Nab3 to granules is PrLD-dependent, there should be a loss of recruitment of GFP-Nab3 to the granule when the PrLD is deleted. Indeed, this is what was observed when the C-terminal 191 amino acids were removed from GFP-Nab3 (“nab3Δ191”, Figs 1B and 2).

We previously characterized an allele of Nab3 in which 24 Q residues distributed across its C-terminal PrLD, were substituted with glutamate [15], thereby reducing the domain’s Q content from 27% to 17%. Collectively these substitutions abrogate *in vitro* amyloid formation by a purified recombinant portion of the domain and cells with this allele as the sole source of Nab3 are not viable [15]. When expressed with an N-terminal GFP-tag, this PrLD-mutant Nab3 showed a strongly reduced ability to form perinuclear granules following glucose restriction (Fig 2). This more targeted alteration of the PrLD (in contrast to deletion of 191 amino acids) confirms a role for the domain in nuclear granule formation.

Nab3 contains a canonical RRM through which it binds nascent RNA during termination [2, 5, 46]. Mutations in the domain impair termination and cell growth. The yeast protein Whi3, which binds RNA through its RRM and contains a Q-rich PrLD, has interactions with RNA important for liquid-like condensate formation in cells [47, 48]. To test if Nab3’s RRM is needed for formation of nuclear granules, a deletion derivative (amino acids 326–371) was generated that lacks its two consensus RNP-motifs. This protein was stably expressed and loss of this domain was lethal (S2 Fig). The GFP-variant of this Nab3 derivative was severely reduced in its ability to form nuclear granules following glucose-starvation (“nab3ΔRRM”, Fig 2). This suggests that, as observed for Whi3, RNA-binding is important for Nab3’s recruitment into granules.

The sequence of Nab3’s PrLD, its propensity to homopolymerize *in vitro*, and the dependence of the protein upon its PrLD to localize to nuclear granules, suggests, although does not prove, that the granule is a dynamic, liquid-like assembly similar to P-bodies. The aliphatic alcohol 1,6-hexanediol has been used as a probe for such assemblies due to its ability to dissolve them; in contrast, structures such as protein aggregates and the cytoskeleton resist solubilization [49, 50]. Cells were glucose starved to form nuclear granules and treated for 30 min with 5% 1,6-hexanediol. This caused a virtually complete loss of granules from cells (“1,6,-hex”, Fig 2), similar to that seen for yeast P-bodies [50].

Since the PrLD sequence isolated from Nab3 can assemble into polymers *in vitro* [14], we asked if a GFP-tagged version of the PrLD itself (GFP-PrLD) could be recruited to nuclear granules. Under glucose-rich conditions, GFP-PrLD was distributed broadly in the cytoplasm and nuclei of cells (S3 Fig). When glucose was withdrawn, the protein formed cytoplasmic aggregates, perhaps at cytoplasmic bodies such as stress granules (S3 Fig). These results indicate that the Nab3 PrLD is not sufficient for recruitment to nuclear granules although surprisingly, the PrLD alone was responsive to glucose starvation in forming cytoplasmic granules.

### Characteristics of the glucose starvation-induced Nab3 granule

**Fig 3:**
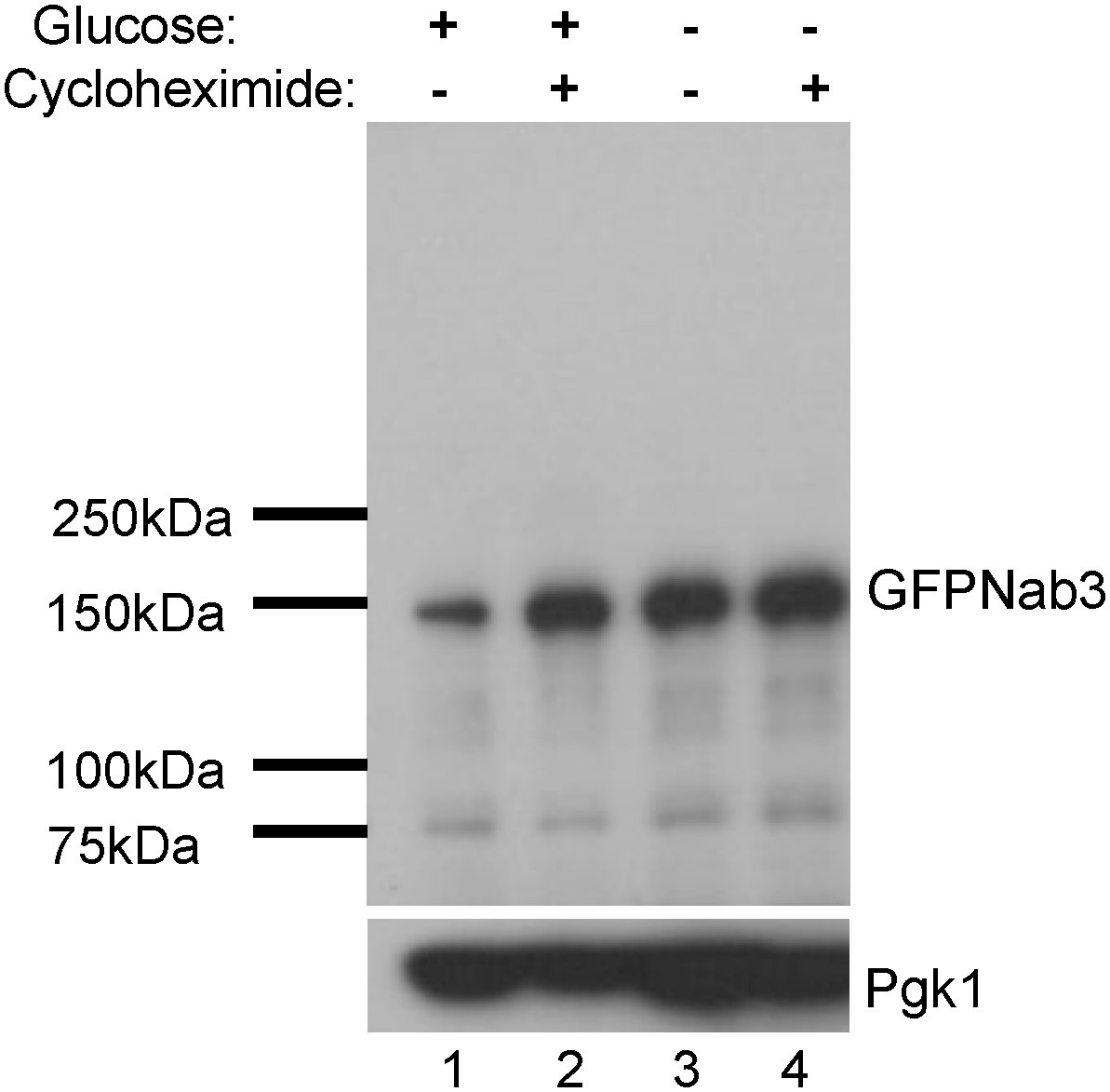
Nab3 levels persist during starvation. To assess the stability of Nab3 under the varying growth conditions, water or cycloheximide (125 μM,) were added as indicated to logarithmically growing DY32309 cells grown in glucose-containing media (lanes 1 and 2). Cells grown in the absence of drug and presence of glucose were washed into glucose-free media, split in two, and one starved culture was treated with cycloheximide for 2 hrs (lanes 3 and 4). Cells were lysed by boiling in electrophoresis sample buffer, subjected to SDS-PAGE, and western blotted using an anti-Nab3 monoclonal antibody as described in Materials and Methods. The filter was stripped and reprobed with anti-Pgk1 as a loading reference.

Corden and co-workers determined that the Nab3-containing granule is distinct from three well characterized ribonucleoprotein structures in yeast: stress granules, P-bodies, and the nucleolus [26]. Other cellular granules are thought to be locations in which substrates are collected for degradation, including insoluble protein deposit (IPOD) and juxtanuclear quality control/intranuclear quality control (JUNQ/INQ) deposits [51, 52]. However, IPOD is a cytoplasmic and perivacuolar depot for terminally segregating proteins, and therefore cannot be the Nab3 granule. JUNQ/INQ has been characterized as a perinuclear granule and in some studies as a nuclear granule in which proteins are prepared for degradation [52]. To test if glucose starvation prompted degradation of Nab3, its levels were measured by western blots of cells treated with cycloheximide to prevent new Nab3 synthesis during glucose deprivation. Nab3 levels were not diminished when glucose was removed and cells were treated with cycloheximide (Fig 3, lane 3 vs lane 4), implying that Nab3’s recruitment to a granule is not a prelude to Nab3 degradation and is not a site of Nab3 turnover. To further explore if the Nab3 nuclear granule was the same as the INQ body, we exploited the finding that INQ formation is dependent upon *BTN2* [52]. Yeast deleted for *BTN2* were transformed with a *LEU2*-marked plasmid expressing GFP-Nab3 and the resulting strain was glucose starved and scored for Nab3 granule formation. *BTN2* null cells could efficiently form Nab3-positive granules (Fig 2). This suggests that the conditional Nab3-containing nuclear granule is not INQ. [53].

**Fig 4:**
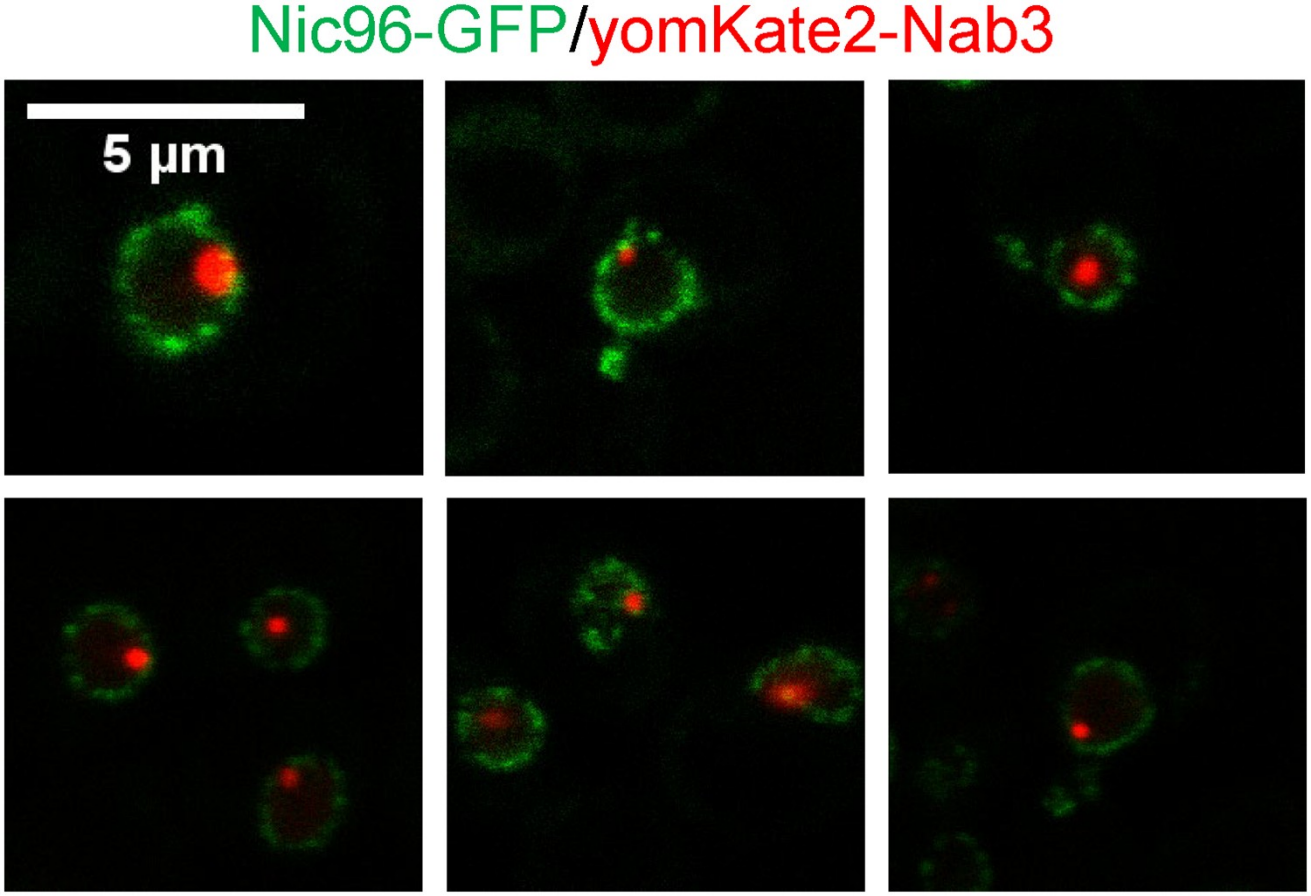
Establishing the cellular location of the Nab3 granule. A yeast strain containing an integrated, GFP-tagged *NIC96* was transformed with an expression vector containing yomKate2-taggedNab3 (DY4538). Cells were washed into SC ura^-^ leu^-^ glucose-free medium, incubated at 30 degrees C for 2hr, and imaged using a confocal microscope. A single Z-plane of representative granule-containing cells is shown.

Previous studies described Nab3 granules as perinuclear, but did not formally test which side of the nuclear membrane the granule was on [26]. To resolve this, we exploited a strain containing a chromosomal copy of GFP-tagged *NIC96*, a linker nucleoporin known to associate with the nuclear pore complex basket and other nucleoporins [54]. Since Nic96 is a component of the nuclear pore, it provided a definitive demarcation of the nuclear periphery [52, 55], allowing assessment of where Nab3 granules reside. This strain was transformed with a plasmid encoding yomKate2-tagged Nab3 and cells were glucose-starved. Images of single Z-axis focal planes across multiple nuclei clearly showed that the Nab3 granule resided within the nucleus as defined by the Nic96 ring, identifying the structure as unequivocally intranuclear (Fig 4).

**Fig 5:**
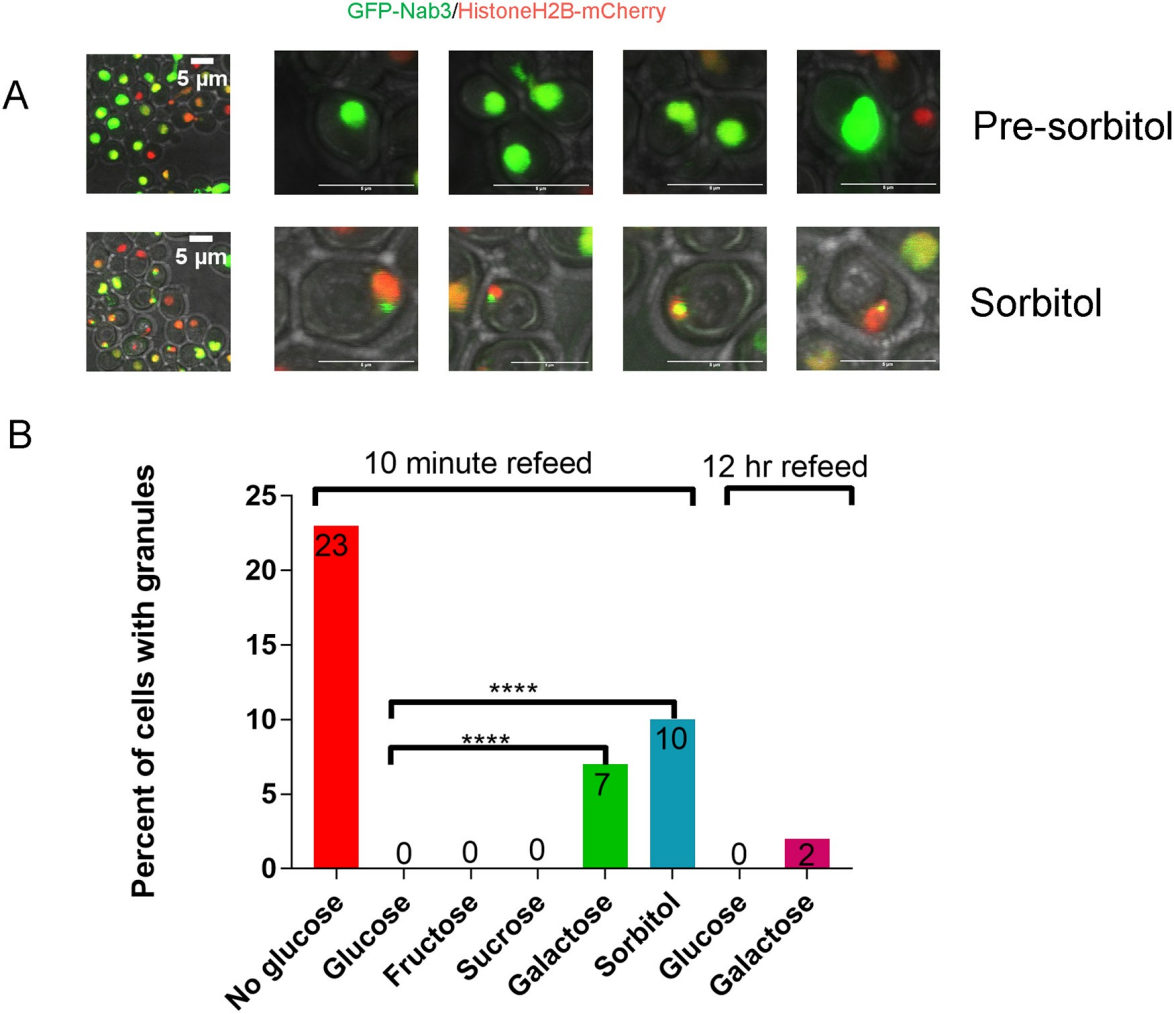
Sorbitol substitution for glucose yields Nab3-granules and does not efficiently reverse granules obtained after glucose starvation. A) DY32309 (GFP-Nab3) cells were grown in glucose, washed, imaged (“Pre-sorbitol”) or incubated for 2 hrs at 30°C with sorbitol before imaging. B) A culture of DY32309 was grown in glucose-containing medium, washed, and incubated for 18 hrs at 30°C in SC ura^-^ leu^-^ lacking glucose. Cells were washed, equivalent numbers were inoculated into media with the indicated sugars (2%), incubated at 30°C for 10 min or 12 hr, as indicated, imaged by confocal microscopy, and the percent of granule-containing cells were quantified. The P-values for a Fisher’s exact test of <0.0001 are indicated by ****.

Resuspending cells in glucose-free medium could represent an osmotic shock [44]. To control for the possibility that such a hypoosmotic challenge is responsible for inducing nuclear granule formation, we substituted equimolar sorbitol for glucose following washout of the latter. Sorbitol acts as an osmotically active solute in growth media to mimic the osmotic environment of glucose-containing media without being a metabolizable carbon source for some yeast [44, 56]. In the strain studied here, sorbitol did not support growth while galactose, sucrose, and fructose could (Panel A in S4 Fig). After 2 hours in sorbitol, nuclear granules were observed as seen above for cells washed out of glucose without a sugar replacement (Fig 5A). The fraction of cells displaying granules was reduced by half relative to the no glucose condition (“sorb, Fig 2), suggesting there could be a partial osmotic component to glucose withdrawal. A similar result of a combined osmotic and metabolic basis for cytoplasmic stress granule formation was observed previously when glucose was replaced with sorbitol [44].

### Quantification and dynamics of the Nab3 nuclear granule

**Fig 6:**
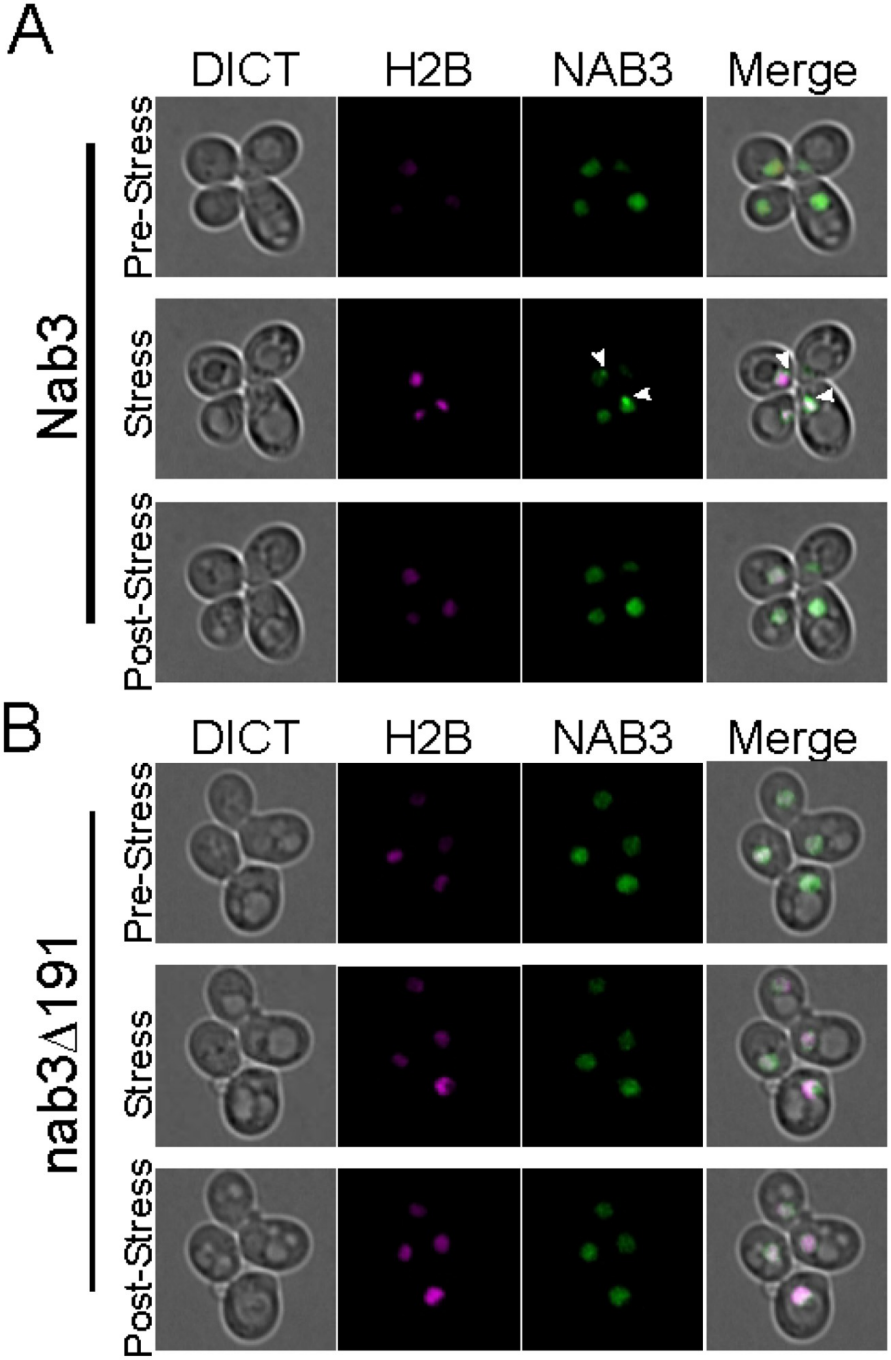
Dynamic recruitment and dissociation of the Nab3 granule. Strains (A) DY32309 (GFP-Nab3) and (B) DY3233 (GFP-nab3Δ191) were examined in a microfluidics chamber using bright field (“DICT”), time-lapse microscopy prior to, during, and after glucose starvation. The nucleus was marked with mCherry-tagged histone H2B(H2B), Nab3 and nab3Δ191 were GFP-tagged(NAB3). Images are single frames of maximum intensity projections from Z-stacks. Cells in glucose-containing medium show a pan-nuclear distribution of Nab3 (“pre-stress” row of A and B). After 2 hr of glucose depletion the Nab3 signal condenses (white arrowheads) in wild type but not in nab3Δ191 (“stress” row of A and B). Ten min following refeeding with glucose-containing medium, the Nab3 granule expands back into a pan-nuclear signal (“post-stress” row) A and B. Time lapse video is available as Supporting Information for both GFP-Nab3 and GFP-Nab3Δ191 expressing strains.

**Fig 7:**
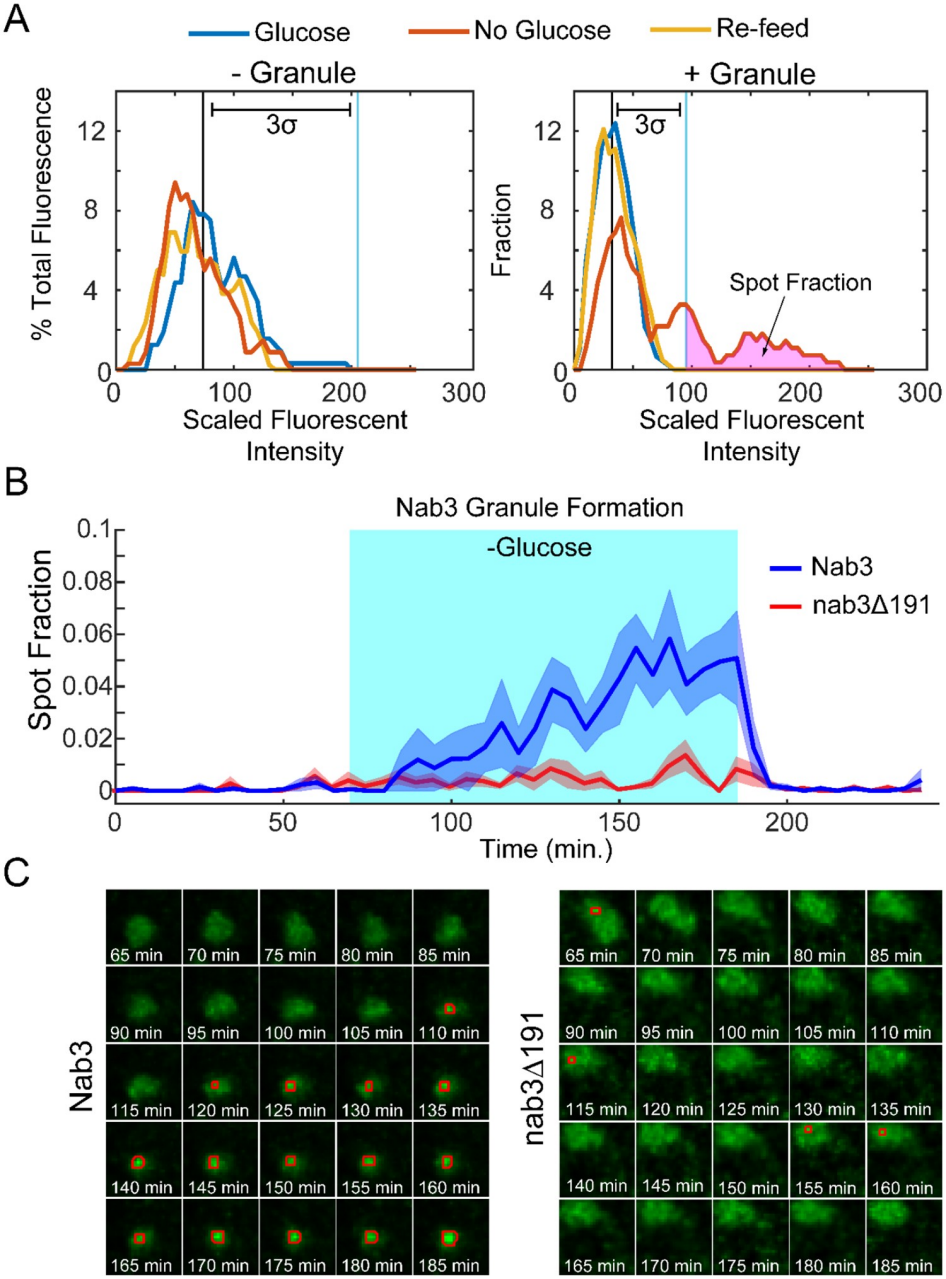
The Nab3 granule is reversible and dependent upon Nab3’s PrLD. A) Illustrative histograms for a single cell scored as lacking (left) and a single cell scored as containing (right), a GFP-Nab3 granule. For each cell, fluorescence over 1 hr growth in glucose (blue peak) was used to calculate a baseline mean and standard deviation. Fluorescent intensity (scale of 0–255, bins of 5) is plotted versus % of fluorescence intensity that each bin is of the total. The amount of fluorescence present more than 3σ above the mean was used to calculate the spot fraction. B) Time course of accumulation of fluorescence in the spot fraction for GFP-Nab3 (blue) and GFP-nab3Δ191 (red). Cells were analyzed by real time fluorescence microscopy in a microfluidic device that allowed the exchange of glucose-containing and glucose-free media. Cells were imaged for 60 min prior in glucose, then switched to no glucose for 2 hours (turquoise area), followed by refeeding with glucose for an hour. The average spot fractions (lines) and standard deviations (shaded areas) for GFP-Nab3 and GFP-nab3Δ191cells were calculated over the time course. C) Time course microscopy of a single nucleus from GFP-Nab3 (left) and GFP-nab3Δ191 (right) starting 5 min after glucose-free conditions (t = 65’). Pixels with fluorescence >3σ for each cell are boxed in red. Cells with Nab3 lacking the PrLD generally did not form granules, but, as seen in this example, those that scored positively show sporadic, small spots, in contrast to the robust and persistent spots formed in wild type Nab3.

Examination of the dynamics of granule formation and relaxation requires the ability to follow a single cell over time while altering the extracellular glucose concentration. To this end, we employed a microfluidics chamber that holds yeast cells stationary while allowing rapid switching of extracellular media [36]. In this way, single live cells can be followed by differential interference contrast bright-field and fluorescence microscopy to monitor Nab3 dynamics before, during, and after glucose starvation. Consistent with the confocal microscopy finding (Figs 1 and 2), a subset of cells in the chamber formed readily observable granules in strains with GFP-tagged wild type *NAB3* following glucose-restriction (Fig 6A, white arrowheads and S1 Movie). In comparison, nab3Δ191 did not form a similar granule in glucose-free media (Fig 6B). To perform an unbiased quantification of granule formation and dynamics, we developed an algorithm based on histogram analysis to quantify the fraction of nuclear fluorescence present in spots. In short, due to its accumulation in a granule, the Nab3 signal is more focused than soluble, nucleoplasmic Nab3. As a baseline, the mean fluorescence intensity peak was calculated during the one hour of growth in glucose prior to starvation for each cell (blue lines, Fig 7A). Pixels with higher Nab3 accumulation show up as a rightward shift in the scaled fluorescent intensity value. We found that the granules contained pixels with fluorescence three standard deviations above the mean. We define the fraction of pixels that meet this criteria as the “spot fraction” (Fig 7A). Using the spot fraction as a metric, cells with GFP-Nab3 (n = 114) were compared to those with GFP-nab3Δ191 (n = 109) as a function of glucose starvation (Fig 7B, shaded area). The average of all cells positive for a spot fraction for both strains are plotted versus time (Fig 7B). This stringent criterion showed that distinct granule-containing and granule-free cells could be resolved (Fig 7A and 7B). Since individual cells could be identified and followed over time, we found that 18% of cells with wild type Nab3 cells formed a stable granule during starvation, consistent with the results seen above using confocal microscopy. Cells that displayed granules kept their granules for the duration of glucose deprivation and those that did not display granules failed to do so for the duration. Strikingly, following the re-addition of glucose, Nab3 granule intensity shifted back to a pan-nuclear baseline distribution within minutes, showing the rapidity of the response to the re-introduction of glucose (Fig 7B and S1 Movie). This is the first demonstration of the reversible nature of the Nab3-containing granule. Importantly, Nab3 lacking its PrLD did not form stable granules (Fig 7B and S2 Movie), recapitulating the observations from confocal microscopy. Once formed in glucose-free conditions, Nab3 granules appeared relatively fixed in location, typically at the nuclear periphery, and persisted until cells were re-fed with glucose (Fig 7B). In contrast, nab3Δ191 formed smaller, lower intensity puncta in 10% of cells. (Fig 7B and 7C). These spots were distinct from granules formed by wild type Nab3 in that they were dim, short lived, and distributed broadly throughout the nucleus, as opposed to the typical, strongly fluorescent, more localized granules seen with GFP-Nab3 (Fig 7C). These granules were not dependent upon exposure to glucose-free media. This again indicates that the PrLD is important for the localization behavior of Nab3 in response to glucose restriction.

**Fig 8:**
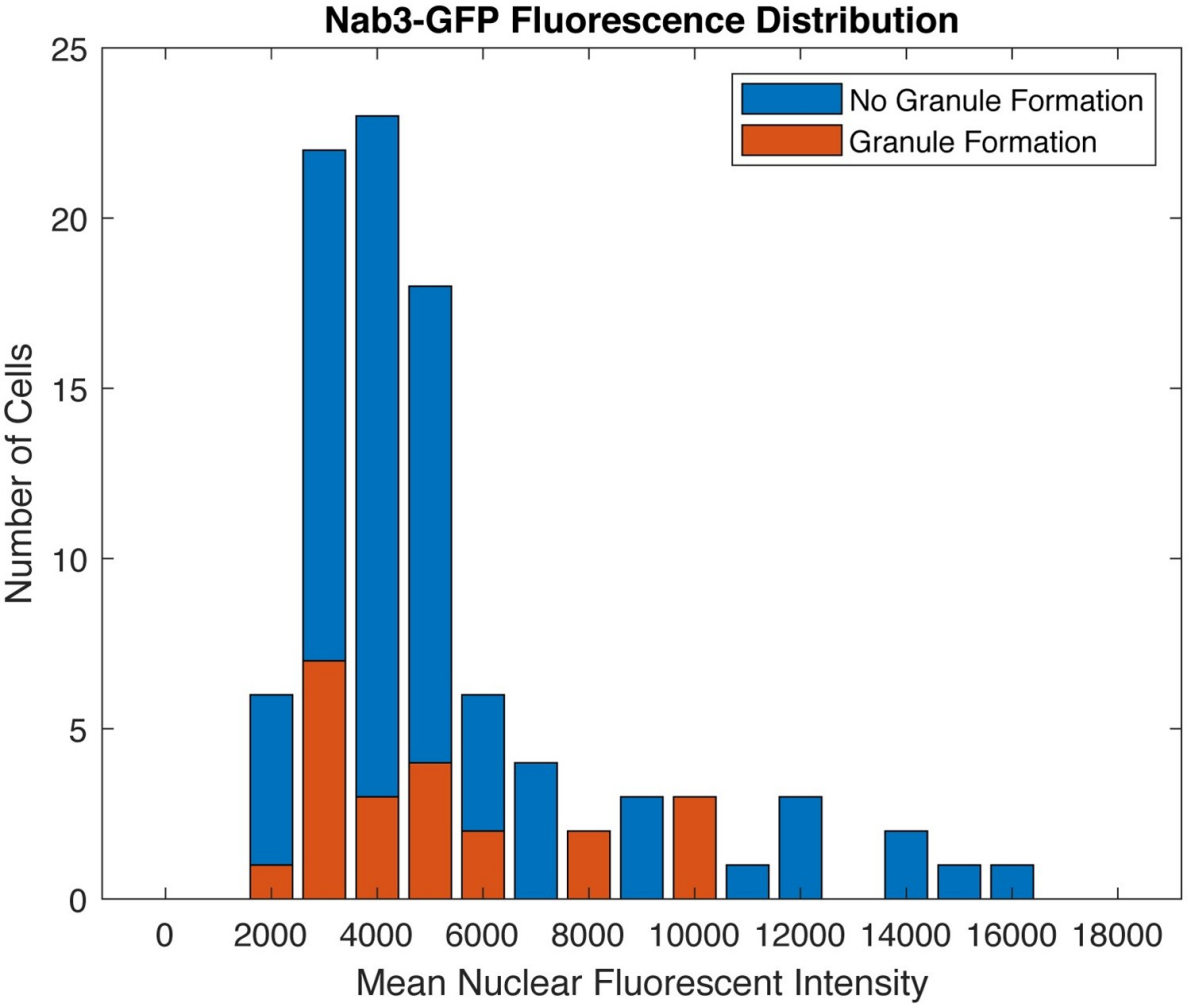
Granule formation is independent of Nab3 expression level. Histogram of the number of cells with a given GFP-Nab3 expression level for both granule-containing and granule-free cells from experiments shown in Fig 7. The GFP-Nab3 expression level represents the average mean fluorescence during the 1 hr in glucose-containing medium, while the cells were scored for granule formation based on the subsequent 2 hr period in the absence of glucose.

A possible complication when expressing heterologous proteins is that granule formation could be triggered by an artificially high concentration of the protein. To address this we examined the mean total GFP-Nab3 fluorescent intensity of nuclei for the 114 cells that would either form granules or fail to form granules during the starvation period (Fig 8). There was no preference for granule formation in high expressor cells indicating that granule formation is not caused merely by ectopic expression of the tagged protein.

Since granule formation correlated with the presence of metabolizable or non-metabolizable sugars in the media, we examined if granule dissolution was also related to sugar ‘usability’. Cells were grown in glucose, washed free of sugar, and incubated at 30°C for 2 hrs to allow granule formation. The culture was then split and cells were re-fed with different sugars. Cells that were re-fed with the metabolizable sugars glucose, fructose, or sucrose, lost their granules after 10 min (Fig 5B). In contrast, the population re-fed with non-metabolizable sorbitol, which did not support growth (Panel A in S4 Fig), retained about half their granules (Fig 5B). Interestingly, cells washed out of glucose and re-fed with galactose showed an unusually long lag period (> 10 hrs) before resuming cell division (Panel B in S4 Fig). A more complete loss of granules required a long incubation (12 hr) with galactose (Fig 5B) consistent with the extended time it took cells to resume proliferation in the presence of this sugar (Panel B in S4 Fig). This correlation between the resumption of growth and magnitude of loss of granules, implies that consumption of the carbon source is important for reversing the effect of glucose-starvation on Nab3 condensation.

We next asked if overall cell viability is compromised following glucose restriction by assaying for colony forming units after 2 hrs without glucose. Cells were washed into glucose-free medium and plated on standard solid medium immediately or after 2 hrs of incubation at 30°C in the continued absence of glucose. Plates were incubated overnight and colony forming units were counted. A similar level of viability was observed for cells before and after glucose starvation indicating there is no deleterious effect of this restrictive regimen (S5 Fig).

### Heterologous PrLDs facilitate Nab3 granule formation

**Fig 9:**
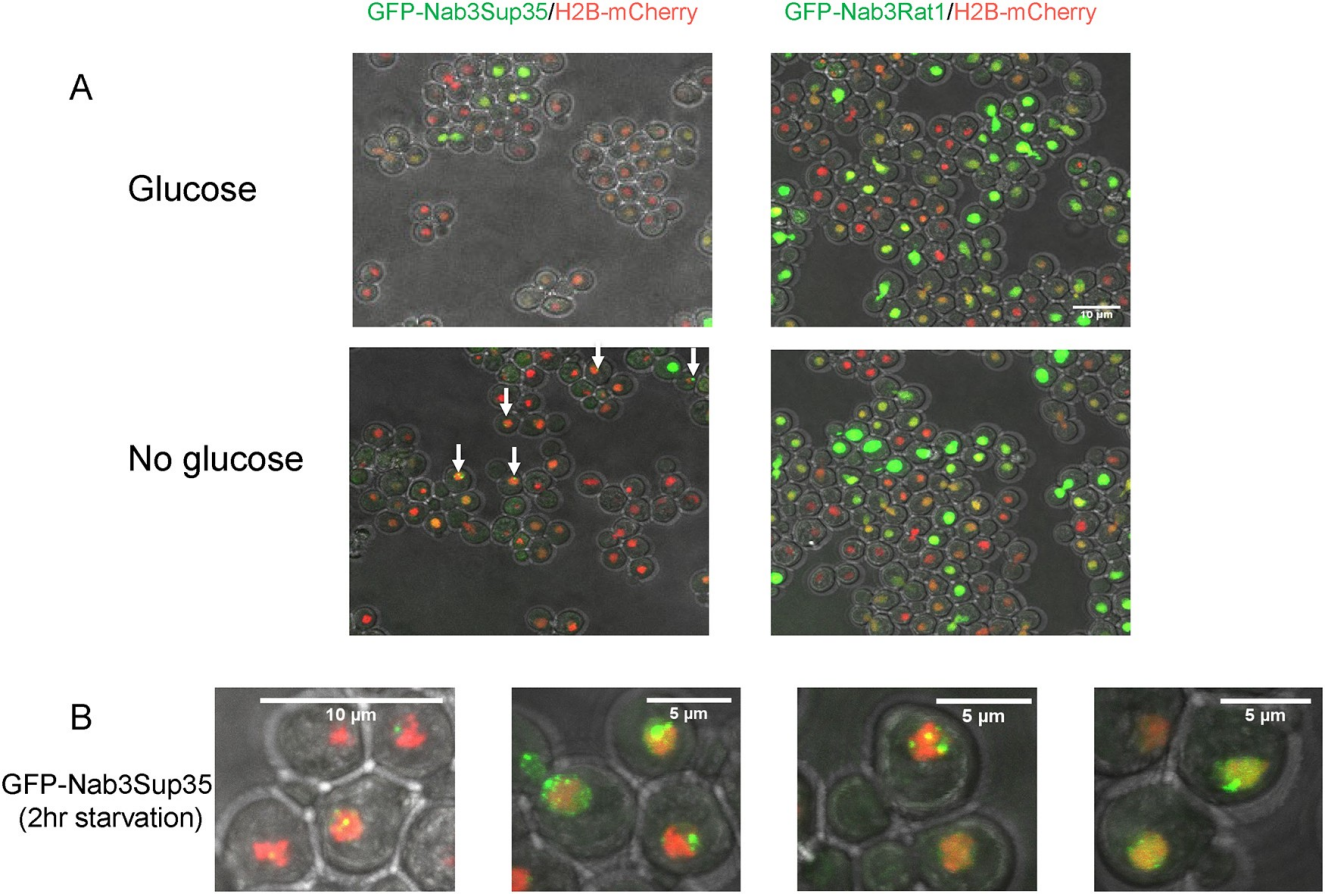
GFP-Nab3Sup35 chimera forms granules following glucose-depletion. **A)** *S*. *cerevisiae* containing an integrated mCherry-tagged histone H2B gene were transformed with a plasmid containing N-terminal GFP-Nab3Sup35 (left; DY4524) or GFP-Nab3Rat1 (right; DY4525). Cells were grown to log phase, washed into SC ura^-^ leu^-^ with or without glucose, incubated at 30°C for 2 hr, and imaged using a confocal microscope. Maximum intensity projections are shown. White arrows show granule-containing cells. B) High resolution images of representative granules in the nuclei of GFPNab3Sup35 containing yeast.

**Fig 10:**
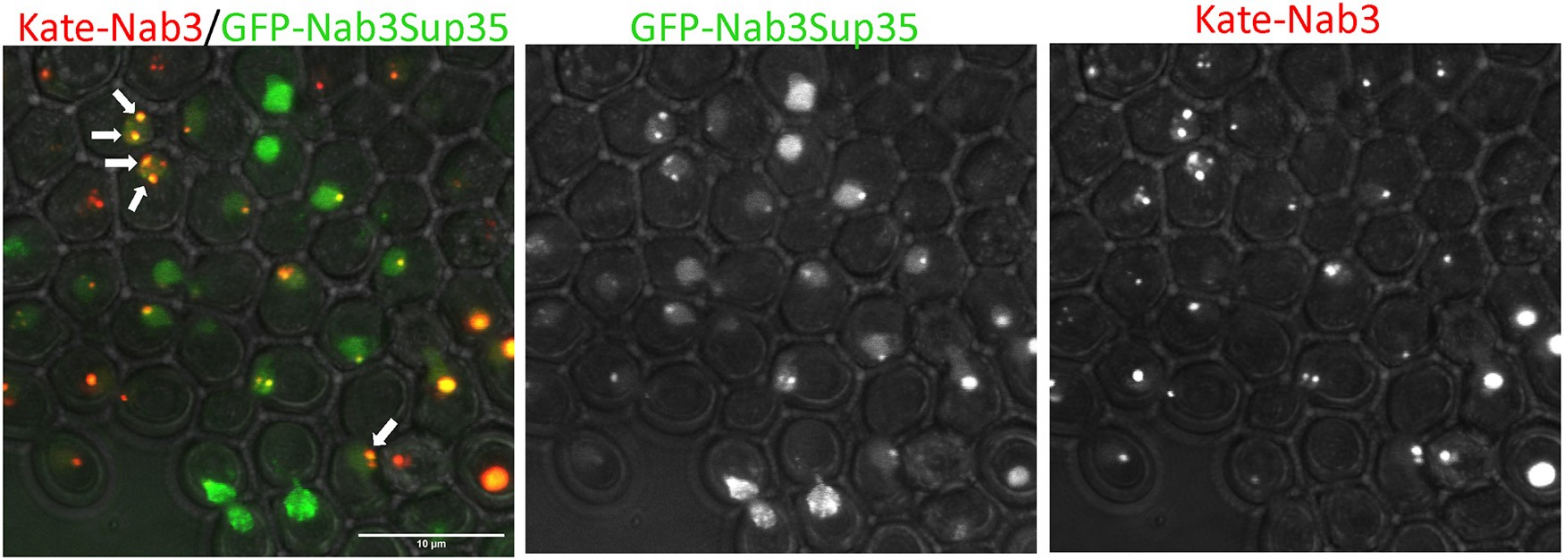
The GFP-Nab3Sup35 chimeric protein localizes to the location that yomKate-Nab3 does, as well as to additional nuclear foci. Strain DY4553 was grown in glucose, washed into glucose-free medium for 2hrs, and imaged by confocal microscopy. Note that the red fluorescent yomKate-Nab3 is almost always encompassed within the green signal (yellow) from GFP-Nab3Sup35 when both reside in the same cell.

We previously showed that some, but not all, heterologous yeast PrLDs can substitute for Nab3’s PrLD thereby conferring viability and termination function onto this transcription factor [16]. We tested whether chimeric Nab3 proteins would relocalize to granules as wild type Nab3 does under glucose starvation. Strains containing untagged, wild type Nab3 were transformed with plasmids expressing GFP-Nab3Rat1, a chimera that does not support cell viability, or GFP-Nab3Sup35, a viable chimera. The chimeric proteins were well-expressed in these strains, though at somewhat reduced levels *versus* wildtype untagged Nab3 (Panel A in S2 Fig). GFP-Nab3Rat1-containing cells exhibited no GFP-positive granules following glucose restriction while GFP-Nab3Sup35 strains were positive for granules (Fig 9A). The granules formed from GFP-Nab3Sup35 were somewhat different from those assembled from GFP-Nab3 with a higher frequency of multiple, lobulated, or fragmented granules apparent when compared to GFP-Nab3 (Fig 9B). To test if the chimeric Nab3Sup35 protein resides in the same granules generated by Nab3, we assembled a strain with GFP-Nab3Sup35 and yomKate-Nab3. This dual labeling strategy showed that Nab3Sup35 is found in the Nab3 granule, as well as outside of the Nab3 granule (Fig 10). The yomKate-Nab3 signal is almost invariably fully encompassed within the GFP-Nab3Sup35 chimera’s when both are found in a single cell. That is, it appears the Nab3Sup35 chimera can successfully localize to the place in which Nab3 is found, as well as becoming focused at additional nuclear locations. This could explain why the Sup35 prion domain can rescue the loss of Nab3’s own PrLD, albeit imperfectly, and suggests that Sup35’s strong prion domain is promiscuous in forming aggregates across cellular compartments.

## Discussion

Evidence is accumulating in favor of a model in which RNA metabolism takes place in compartments assembled through liquid-liquid phase separation by RNA-binding proteins that contain low complexity, prion-like domains [17, 18, 39]. Many cytoplasmic granules are inducible such as P-bodies and stress granules. Our results suggest that a compartment that harbors the hnRNP-like Nab3 termination factor, may employ such a mechanism in the nucleus. The functional consequence of this subnuclear granule is not clear, although its inducible assembly serves as a valuable paradigm to study the rearrangement of these RNA binding proteins. Our evidence is congruent with the idea that the NNS system is involved in the transcriptome changes observed following glucose starvation [26, 57, 58].

The Nab3-Nrd1-containing granule was first identified [26] as a novel structure in or near the nucleus that resembled an RNA quality control site seen in cells with mutated RNA processing factors [59]. Here we observe the reversible induction of Nab3 granules in a PrLD-dependent manner. A caveat of this analysis is that we have examined a GFP-fusion version of the protein and not the native version of Nab3. Nevertheless, the signature low complexity domain and its well-established homopolymerization properties suggest that the native Nrd1-Nab3 termination machinery forms a functionally important compartment for RNA metabolism. This process may be akin to ‘transcription factories’ at which one or more transcribed genes congregate and where the many steps of pre-mRNA synthesis may take place in a concerted fashion [60]. In metazoan cells, other nuclear compartments that perform a more limited task are so-called nuclear speckles which are enriched for splicing factors [61] or the speckles formed by the elongation factor P-TEFb, a kinase that associates with RNA polymerase II [22]. The cyclin T1 subunit of P-TEFb employs a low complexity domain to associate with RNA polymerase II through a phase separation mechanism [22]. Along this line of thinking, the Nab3-Nrd1 granule may form through phase separation at sites in need of its termination activity.

An alternative possibility is that the Nab3 granule may be a location for the storage of inactive proteins that can then reorganize when a physiological stimulus is detected, in this case, the return of sugar to the growth medium. Perhaps the granule forms at a specific portion of the cell cycle. In any event, we postulate that detection of the granule after glucose starvation is a special case of Nab3 assembly, and that during normal vegetative growth, Nab3 and Nrd1 regularly carry out routine transcription termination as a higher order assembly in an RNA polymerase II-containing termination complex. The form they take during vegetative growth would be submicroscopic. This idea is supported by evidence of the self-assembly property of Nab3 *in vivo* and *in vitro* [12, 14, 62] and is derived from an early model that multiple Nab3-Nrd1 complexes are loaded onto nascent RNA [63]. It is possible that Nrd1 also forms oligomers with itself during termination, as it bears a low complexity domain [13], a recombinant version of which forms amyloid filaments [14]. These two proteins also dimerize efficiently through domains outside of their PrLDs [63], leading to the possibility of large Nab3-Nrd1 co-polymers.

The Nab3 rearrangement noted here was detected by confocal microscopy as well as differential interference contrast microscopy using flow chambers in which single cells can be observed over time. The dynamic Nab3-Nrd1 granules are similar to the glucose deprivation-induced distribution of cytoplasmic stress granules and processing-bodies seen in the yeast cytoplasm [38, 44, 45]. Interestingly, and in contrast to Nab3, yeast Hrp1 migrates from the nucleus to cytoplasmic stress granules following glucose starvation [38]. Hrp1 is a polyadenylation-coupled transcription termination factor that bears a PrLD and assembles into amyloid polymers *in vitro* [13, 16, 64]. Here we show the Nab3 granule is clearly intranuclear and that its formation is reversible. It is also notable that formation of the granule takes hours whereas its dissolution takes minutes. The biochemical changes that regulate this process are an obvious next important question and are the focus of many studies into ribonucleoprotein granule assembly through liquid-liquid phase separation. In this case, the stimulus for granule formation derives from the cell’s access to a usable carbon source. Nutrient depletion and the Ras/protein kinase A pathway have been genetically linked to Nab3 and Nrd1 [26]. The biochemical details of how directly this signaling pathway is connected to granule formation remains to be defined.

Another important question is what other macromolecules may be harbored in this nuclear granule. It does not appear to be a location at which the Nab3 protein is degraded, such as that seen for proteins that aggregate in the JNQ and INQ compartments [52, 53]. If it is a site of active termination, then we predict RNA polymerase II and nascent RNA will co-localize with Nab3. Resolving the role of this cellular compartment and how its assembly is regulated will require further examination.

## Supporting information

S1 Fig Granule formation in a yeast strain in which the *GFP-NAB3* fusion was integrated into the chromosome. Strain DY4543 was grown in glucose washed into the same medium (left) or into glucose-free medium (right) as described in Materials and Methods and imaged using confocal microscopy to detect fluorescent GFP-Nab3. In the presence of glucose no nuclei show granules. Granules (a subset of which are indicated with white arrows) were found in 18% (56 of 319) of the nuclei in cells starved for glucose.
(TIF)

S2 Fig Expression of GFP-Nab3 mutant and GFP-Nab3 chimeric proteins. A) Log phase cultures of DY32309 (lanes 1 & 4), DY4524 (lane 2), DY4525 (lane 3), and DY4551 (lane 5), DY3233 (lane 6) and DY4546 (lane 7) were collected, lysed in boiling electrophoresis sample buffer and subjected to SDS-PAGE (5%, lanes 1–3 and 8%, lanes 4–5; 6% lanes 6–7) and western blotting using an anti-Nab3 antibody as described in Materials and Methods. The Q→E variant of Nab3 runs anomalously slowly as seen previously [15]. B) Strains with wildtype *NAB3* on a *URA3*-marked plasmid and a *LEU2*-marked plasmid with either GFP-tagged Nab3 (DY32309) or GFP-tagged Nab3ΔRRM (DY4551) were grown overnight in SC leu^-^ glucose media, struck to SC-FOA, incubated at 30 degrees C for 48hrs then imaged. The strain with Nab3 deleted for its RRM as the only source of Nab3 was not viable.
(TIF)

S3 Fig Nab3’s 191-amino acid PrLD forms cytoplasmic granules after glucose restriction. *S*. *cerevisiae* containing an integrated mCherry-tagged histone H2B was transformed with a plasmid containing GFP fused to the 191-amino acid Nab3 PrLD (DY4549). Cells were washed into SC ura^-^ leu^-^ glucose-free medium, incubated at 30°C for 2hr and a single Z plane was imaged using confocal microscopy.
(TIF)

S4 Fig Growth of yeast on various sugars. A) DY32309 cultures were grown overnight to OD_600_ of approximately 0.4, washed free of glucose, and inoculated into media with 2% of the indicated sugar. Cultures were grown at 30°C for 18 hrs before photographing. B) Cultures of DY32309 were grown in glucose-containing medium to OD_600_ of approximately 0.4, washed, and grown in glucose-free medium for 2 hr at 30°C. Equivalent numbers of cells were inoculated into glucose-, galactose-, or sorbitol (2%)-containing medium and returned to 30°C. OD_600_ was scored in triplicate biological repeats. The mean and standard deviations were plotted.
(TIF)

S5 Fig Survivability post glucose-depletion. Three separate cultures (biological replicates) were grown to log phase in SC ura^-^ leu^-^ with glucose and washed into glucose-free medium. Equivalent cell numbers were plated onto glucose-containing medium immediately or after 2 hrs in glucose-free medium at 30 degrees C. Colonies (viable cells) were counted and plotted. Error bars are the standard error of the mean (SEM). For “zero point Nab3” mean ± SEM = 215 ± 17.2, n = 3, for “2 hour Nab3” mean ± SEM = 231.7 ± 6.0, n = 3.
(TIF)

S1 Movie Video of strain with GFP-Nab3 during glucose starvation. Strain DY32309 (GFP-Nab3) was examined in a microfluidics chamber using bright field (“DICT”), time-lapse microscopy prior to, during, and after glucose starvation. The nucleus was marked with mCherry-tagged histone H2B, Nab3 was GFP-tagged. The movie is a time lapse of one hour in glucose containing media, two hours in glucose free media followed by one hour upon glucose re-addition. A subset of GFP-Nab3 containing yeast exhibit GFP-Nab3 granule formation upon glucose removal that is rapidly reversed upon glucose re-addition.
(3GP)

S2 Movie Video of strain with GFP-Nab3Δ191 during glucose starvation. Strain DY3233 (GFP-Nab3Δ191) was examined in a microfluidics chamber using bright field (“DICT”), time-lapse microscopy prior to, during, and after glucose starvation. The nucleus was marked with mCherry-tagged histone H2B, Nab3**Δ**191 was GFP-tagged. The movie is a time lapse of one hour in glucose containing media, two hours in glucose free media followed by one hour upon glucose re-addition. GFP-Nab3 Δ191 exhibits a pan nuclear distribution throughout the time course.
(3GP)
