# Supplementary figures and images for: Nab3’s localization to a nuclear granule in response to nutrient deprivation is determined by its essential prion-like domain
Source: PLoS One. 2018 Dec 17;13(12):e0209195. doi: 10.1371/journal.pone.0209195 (PMC6296506; doi:10.1371/journal.pone.0209195)

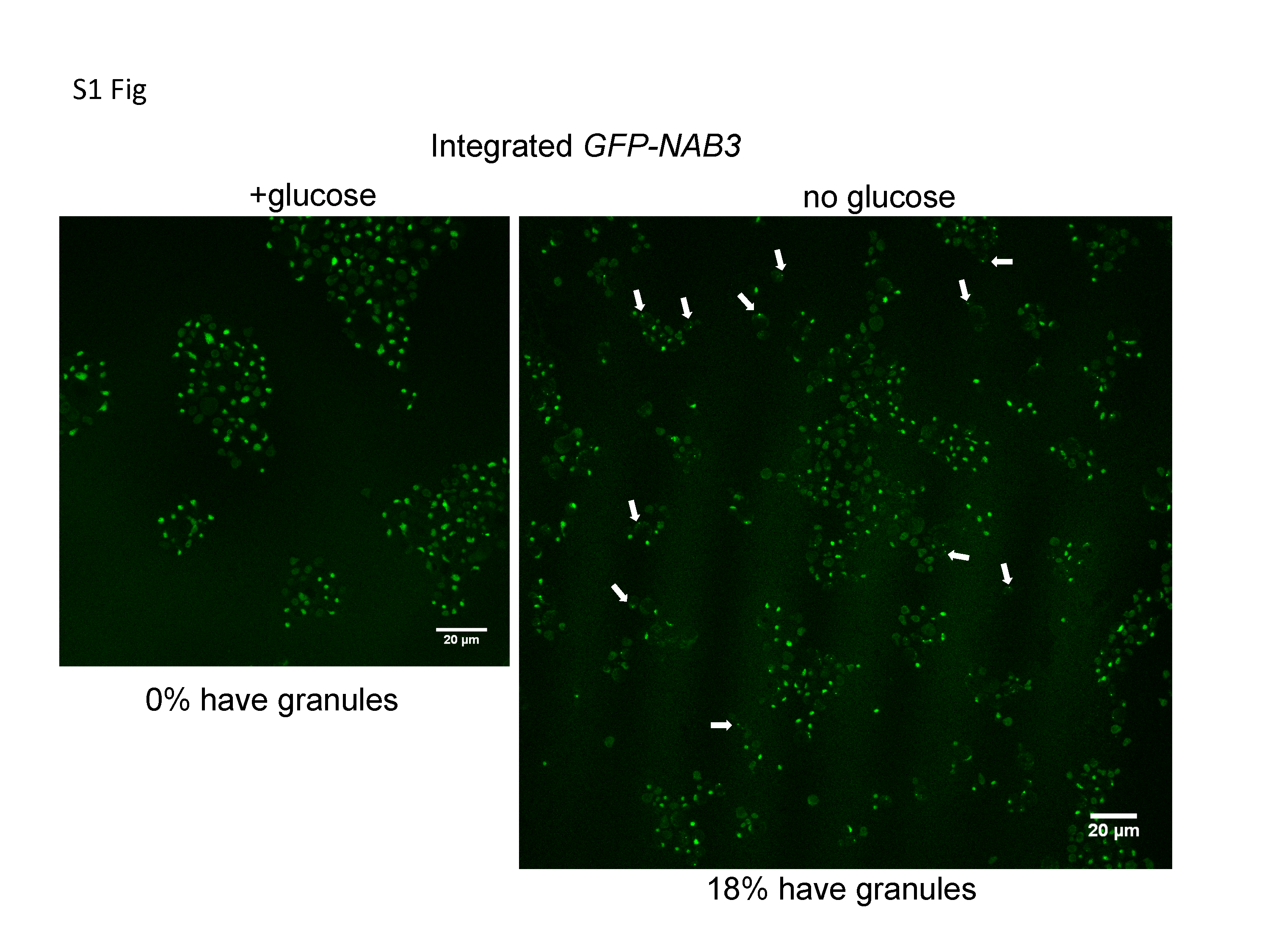

Supplement: S1 Fig — Strain DY4543 was grown in glucose washed into the same medium (left) or into glucose-free medium (right) as described in Materials and Methods and imaged using confocal microscopy to detect fluorescent GFP-Nab3. In the presence of glucose no nuclei show granules. Granules (a subset of which are indicated with white arrows) were found in 18% (56 of 319) of the nuclei in cells starved for glucose. (TIF) [file pone.0209195.s001.tif]

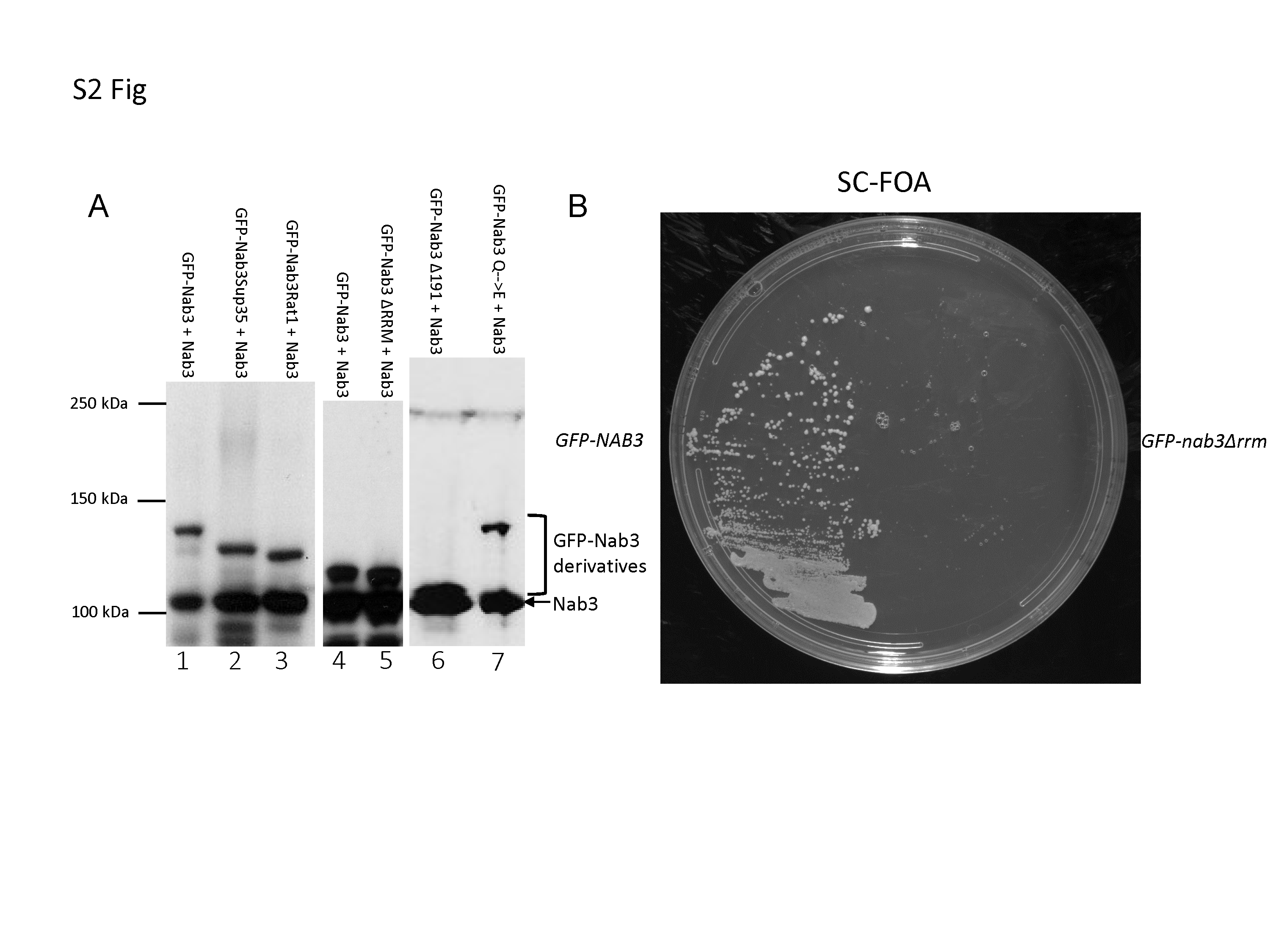

Supplement: S2 Fig — A) Log phase cultures of DY32309 (lanes 1 & 4), DY4524 (lane 2), DY4525 (lane 3), and DY4551 (lane 5), DY3233 (lane 6) and DY4546 (lane 7) were collected, lysed in boiling electrophoresis sample buffer and subjected to SDS-PAGE (5%, lanes 1–3 and 8%, lanes 4–5; 6% lanes 6–7) and western blotting using an anti-Nab3 antibody as described in Materials and Methods. The Q→E variant of Nab3 runs anomalously slowly as seen previously [15]. B) Strains with wildtype NAB3 on a URA3-marked plasmid and a LEU2-marked plasmid with either GFP-tagged Nab3 (DY32309) or GFP-tagged Nab3ΔRRM (DY4551) were grown overnight in SC leu- glucose media, struck to SC-FOA, incubated at 30 degrees C for 48hrs then imaged. The strain with Nab3 deleted for its RRM as the only source of Nab3 was not viable. (TIF) [file pone.0209195.s002.tif]

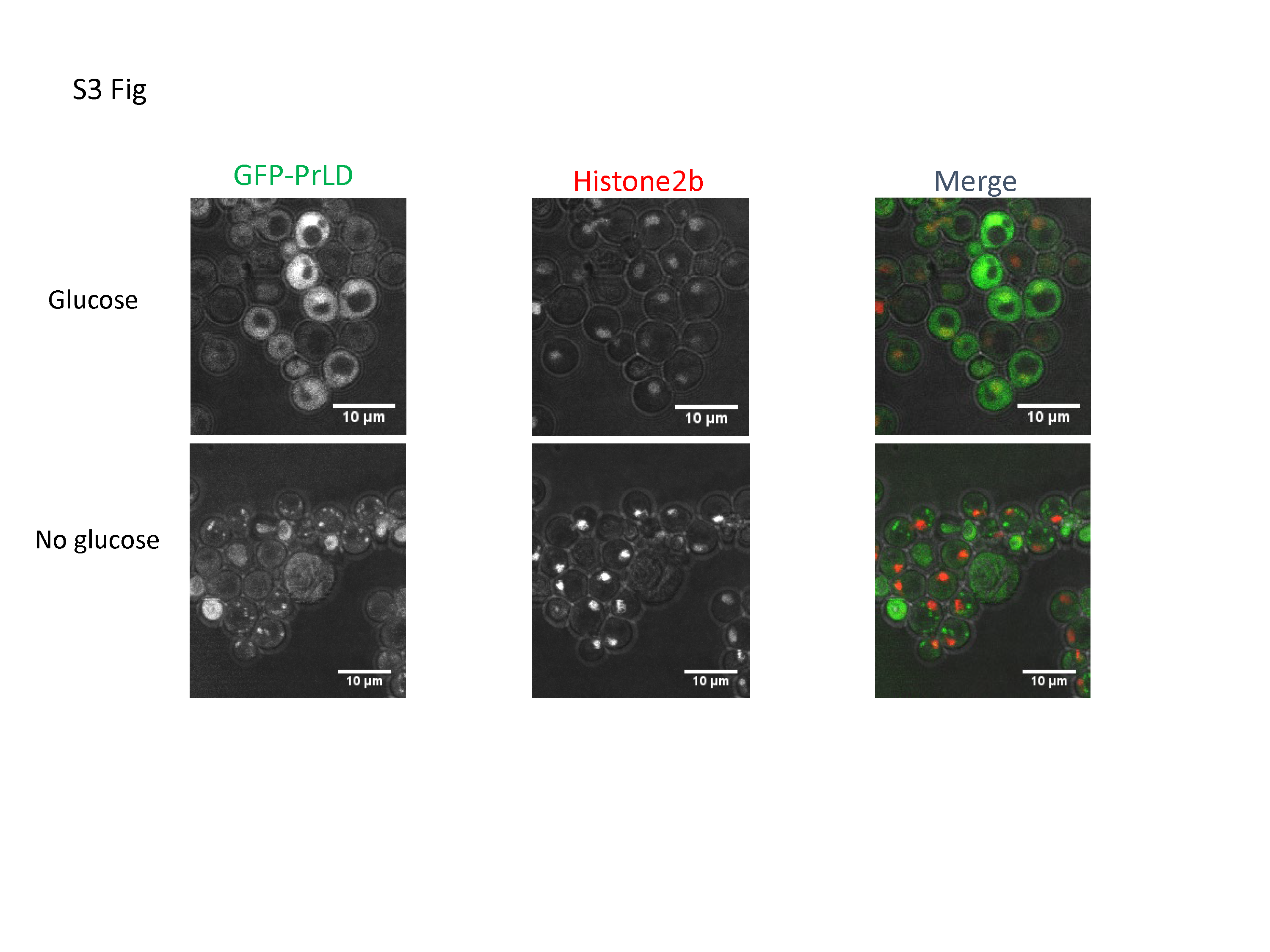

Supplement: S3 Fig — S. cerevisiae containing an integrated mCherry-tagged histone H2B was transformed with a plasmid containing GFP fused to the 191-amino acid Nab3 PrLD (DY4549). Cells were washed into SC ura- leu- glucose-free medium, incubated at 30°C for 2hr and a single Z plane was imaged using confocal microscopy. (TIF) [file pone.0209195.s003.tif]

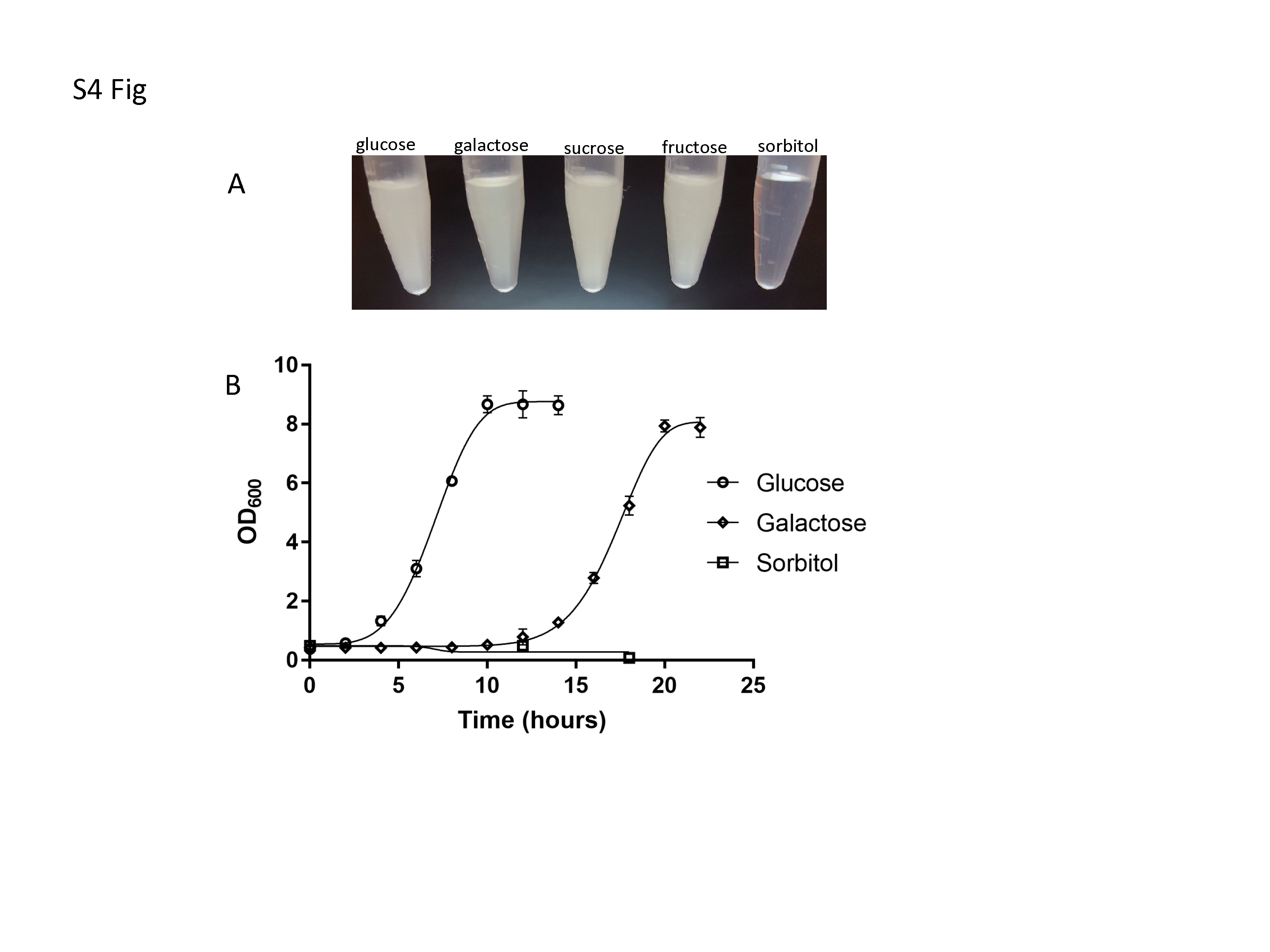

Supplement: S4 Fig — A) DY32309 cultures were grown overnight to OD600 of approximately 0.4, washed free of glucose, and inoculated into media with 2% of the indicated sugar. Cultures were grown at 30°C for 18 hrs before photographing. B) Cultures of DY32309 were grown in glucose-containing medium to OD600 of approximately 0.4, washed, and grown in glucose-free medium for 2 hr at 30°C. Equivalent numbers of cells were inoculated into glucose-, galactose-, or sorbitol (2%)-containing medium and returned to 30°C. OD600 was scored in triplicate biological repeats. The mean and standard deviations were plotted. (TIF) [file pone.0209195.s004.tif]

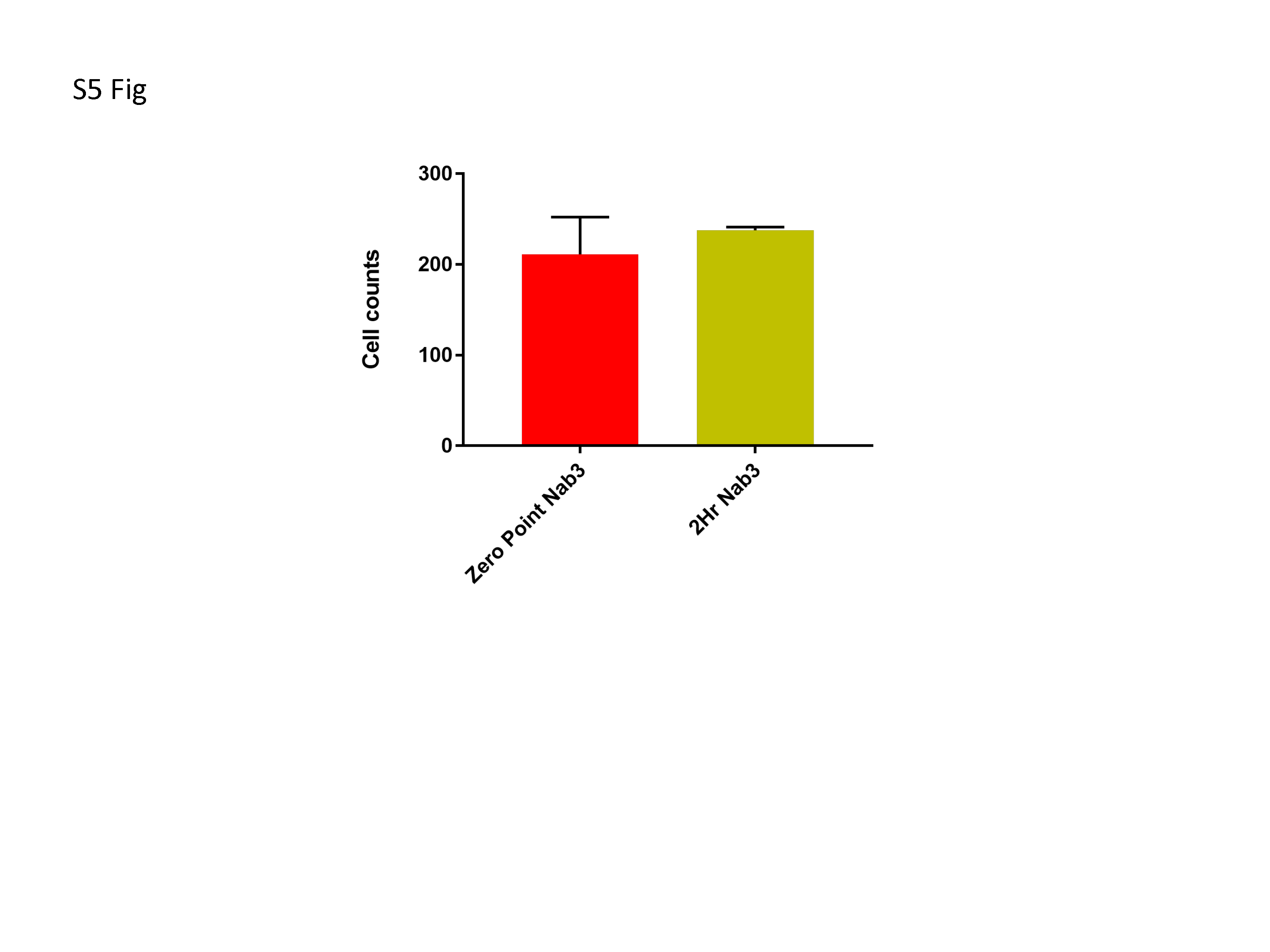

Supplement: S5 Fig — Three separate cultures (biological replicates) were grown to log phase in SC ura- leu- with glucose and washed into glucose-free medium. Equivalent cell numbers were plated onto glucose-containing medium immediately or after 2 hrs in glucose-free medium at 30 degrees C. Colonies (viable cells) were counted and plotted. Error bars are the standard error of the mean (SEM). For “zero point Nab3” mean ± SEM = 215 ± 17.2, n = 3, for “2 hour Nab3” mean ± SEM = 231.7 ± 6.0, n = 3. (TIF) [file pone.0209195.s005.tif]
